# Exposure to plastic debris alters expression of biomineralization, immune, and stress-related genes in the eastern oyster (*Crassostrea virginica*)

**DOI:** 10.1371/journal.pone.0319165

**Published:** 2025-04-29

**Authors:** Laura E. Eierman, Jacob Landis

**Affiliations:** 1 Biological Sciences Department, SUNY Cortland, Cortland, New York, United States of America; 2 School of Integrative Plant Science, Section of Plant Biology and the L.H. Bailey Hortorium, Cornell University, Ithaca, New York, United States of America; Laboratoire de Biologie du Développement de Villefranche-sur-Mer, FRANCE

## Abstract

The degradation of marine plastic debris poses a threat to organisms by fragmenting into micro- and nano-scale pieces and releasing a complex chemical leachate into the water. Numerous studies have investigated harms from plastic pollution such as microplastic ingestion and exposure to single chemicals. However, few studies have examined the holistic threat of plastic exposure and the synergistic impacts of chemical mixtures. The objective of this study was to measure changes in gene expression of gill and gonadal tissue of the eastern oyster (*Crassostrea virginica*) in response to plastic debris exposure during their first year, using RNA-seq to explore multiple types of physiological responses. Shell and polyethylene terephthalate plastic were used as substrate for the metamorphosis of larval oysters in a settlement tank. Substrate pieces were then transferred to metal cages and outplanted in pairs – shell cage and plastic cage – onto restoration reefs in the St. Mary’s River, Maryland, USA. After 10 months of growth, the oysters were collected, gill and gonadal tissue removed, and sex identified. The tissues of six oysters from each sex and substrate type were then analyzed in RNA-seq. Both gill and gonadal tissue samples had altered expression of immune and stress-response genes in response to plastic exposure. Genes upregulated in response to plastic were enriched for gene ontology functions of proteolysis and fibrinolysis. Downregulated genes were involved in shell biomineralization and growth. One male oyster exposed to plastic had “feminized” gene expression patterns despite developing mature sperm, suggesting plastic leachate can alter gene expression and shift protandric individuals to develop as females. Plastic pollution may therefore reduce shell growth, initiate immune and stress responses, alter sex differentiation, and impact reproductive output of eastern oysters through changes in transcription.

## Introduction

The trillions of plastic pieces floating in the pelagic ocean are well documented, from large scale studies [1–6] to studies on local waterways such as Chesapeake Bay tributaries [7]. In addition to the plastic afloat at or near the sea surface, large quantities of plastics have been observed in benthic habitats [8,9] and the deep sea [10–12]. In the presence of seawater, virgin plastic undergoes several physical and chemical transformations. First, the components of the plastic material can leach into the water through the release of unincorporated surface monomers and the movement of additives such as plasticizers and flame retardants through the pores of plastic [13–16]. Example additives include bisphenol A (BPA), phthalates, and alkylphenols that degrade easily to nonylphenol (NP), all of which are known endocrine disruptors [16]. Second, the plastic polymer can degrade in seawater, a process initiated by UV radiation and oxygen and by hydrolysis for plastics such as polyethylene terephthalate (PET) [13] Third, plastics can sorb hydrophobic organic contaminants (HOCs) such as polychlorinated biphenyls (PCBs) and 2,20 -bis(p-chlorophenyl)-1,1-trichloroethane (DDT) [15–19] as well as metals, particularly heavy metals such as cadmium and lead [20–24]. The circulation of plastic pieces in the oceans transports HOCs globally [15] and expose organisms to HOCs [25]. Fourth, macro- and mesoplastics can fragment, progressively becoming smaller and breaking into additional pieces over time [26–29]. Fragmentation forms secondary microplastics, which are distinct from the primary microplastics that directly enter the environment as pre-existing small particles [30]. Microplastics can continue to fragment into nanoplastics with larger relative surface areas that increase the sorption of pollutants and the reactivity of their surfaces [21,31–34]. These plastics are also taken up by cells through endocytosis as well as passive penetration [34,35]. Oceanic bacteria, including pathogenic species like *Vibrio* sp., can colonize surfaces and the cracks within the plastics, contributing to the fragmentation process [36,37]. The breakdown of plastic marine debris therefore poses a complex, multi-faceted risk to marine organisms and requires a comprehensive approach to fully understand potential harm.

Risk assessment of microplastics has dominated recent research on plastic pollution. Multiple studies have built a foundational understanding on the prevalence and types of microplastic ingested by bivalves worldwide [38–44] as well as the role of adherence in contributing to observations of microplastics in soft body tissues [45]. Laboratory ingestion studies using virgin, primary microplastics mostly made of polystyrene have found a range of negative impacts on digestion in bivalves including reduced clearance rates [46,47], reduced filtration rates [48], decreased total energy and protein content [49], and altered activity of digestive enzymes [50], all of which could impact the ability of bivalves to acquire energy. Bivalves also demonstrate an increase in oxidative stress and antioxidant upregulation [51,52], with notable differences between sexes in susceptibility to stress at low microplastic concentrations [48]. Microplastic exposure has also led to reduced fecundity, larval survival and development, and energy uptake as well as increased inflammatory immune responses [53,54]. The broad range of physiological responses indicates that microplastic ingestion is altering multiple distinct functions within bivalves.

Fewer studies have investigated the physiological impacts of chemical exposure from plastic leachate [55,56]. Several additives such as BPA and phthalates are endocrine disruptors, and many studies have focused on the impacts of these individual chemicals [55]. In mussel species, BPA altered gene transcription and shell formation during early embryo development [57], destabilized the lysosome membrane of hemocytes [58], damaged ovarian follicles and induced transcription of phospho-proteins in females*,* and induced spawning in both sexes [59]. In *Crassostrea gigas*, BPA exposure was more likely to be fatal to males than females [60]. In mussels, diallyl phthalate caused males to spawn and downregulated the expression of phospho-proteins in both sexes [59]. BPA and diallyl phthalate increased micronuclei frequency in the gill tissue, and diallyl phthalate increased fragment apopotic cells, both responses indicative of genotoxic and cytotoxic contamination [61]. Plastic additives are clearly detrimental to bivalves, but little is known about the comprehensive impacts of the diverse chemicals leaching as plastic degrades in the marine environment.

Studies of wild bivalves have found the accumulation of plastic additives such as biphenyl S, biphenyl F, bis (2-ethylhexyl) phthalate, and other phthalates in their soft tissues [41,42] indicating that they are exposed to and impacted by plastic leachate. Research into whole plastic debris, including those labeled BPA-free such as PET [62], suggest they leach a variety of chemicals, many of which act as endocrine disruptors [63–65]. An investigation into the leachate from a PET pharmaceutical bottle identified over 130 chemicals including heavy metal additives used in the synthesis of plastic [66]. Similarly, estrogenic contamination was found in 60% of the water tested in PET bottles [65]. With multiple chemicals interacting, it is possible for these mixtures to have a synergistic effect [15]. However, few studies have directly studied the impact of such chemically complex leachates. These studies have found a broad range of negative impacts on mollusks from direct plastic leachate including increased reactive oxygen species in tissues, decreased expression of genes responsive to oxidative stress [66], decreased survival and abnormal growth and development in larvae and juveniles [67–69], sex ratios skewed female [69], increased reproductive output [65], and changes in behavior such as reduced motility and disrupted responses to predatory cues [70,71].

Given the multidimensional risk posed by plastics, studies have begun to investigate the impact of both microplastics and chemical exposure, through either the sorbed chemicals on ingested microplastics or the leachate released during ingestion of, or exposure to, microplastics [67,72–74]. In a unique study on *Tegillerea granose*, microplastics and BPA were studied both separately and combined, with exposure to both yielding larger effects of neurotoxicity than either did individually [75], suggesting synergistic effects of plastic fragments and chemical leachates. As our understanding of the complex threat posed by plastic debris grows, there has been a building call for more work addressing the impacts of plastic leachate on marine organisms at environmentally relevant concentrations and mixtures [55,56,76]. With both microplastic and chemical leachate exposure causing such wide-ranging impacts, a holistic approach is needed to determine the full danger of plastic in marine systems [15]. Additionally, the need for understanding the impacts on keystone species and ecosystem engineers has explicitly been identified [76].

The eastern oyster (*Crassostrea virginica*) is a keystone species that builds reefs in estuaries along the western North Atlantic. They regulate water quality, create habitat for over 300 species, and provide critical ecosystem services [77–80]. However, over 90% of oyster reefs have been lost in North America since their peak harvest days in the 1800s [77,81,82]. The eastern oyster is a valuable species for investigating how plastic debris may impact sexual development. They are protandric hermaphrodites that undergo gametogenesis each year [83]. Evidence suggests a simple genetic model underlying three “sexes”: oysters that are male, oysters that are female, and oysters that begin male and change to female [84]. The hermaphroditic genotype may also be able to change back to males [83] and in rare instances, the oysters may produce sperm and eggs at the same time [84]. Sex differentiation during gametogenesis is due to changes in gene expression [85–87]. By studying plastic exposure in the eastern oyster, we can measure changes in gene expression in a hermaphroditic species in response to the multi-faceted dangers presented by plastic debris.

The objective of our study is to measure changes in gene expression in gill and gonadal tissue of first-year eastern oysters reared on PET substrate in comparison to those raised on natural shell substrate as a control. Using plastic as a treatment substrate ensures chronic exposure to plastic as it degrades and fragments in the marine system, presumably releasing both secondary microplastics and leachate. We measured differential gene expression between sexes and between substrates in both tissue types to identify genes and gene functions impacted by holistic plastic exposure in natural waters. We hypothesized that plastic exposure increases the expression of genes involved in stress response such as oxidative stress and genotoxic stress and in immune function in both gill and gonadal tissue. We also hypothesized an increase expression of genes involved in female gonad and oocyte development in gonadal tissue of both sexes in response to plastic exposure due to endocrine disruption. Identification of differentially expressed genes and enriched gene functions will provide foundational information on the transcriptional underpinnings of the diverse impacts of plastic pollution on marine organisms, paving the way for more targeted investigations into the how plastic alters physiological processes.

## Methods

### Oyster treatment

Two substrates were selected for settlement of larval oysters. Oyster shell used for restoration reef construction that had been cleaned and left in the sun for a year was the control substrate. Plastic beverage bottles made of PET were used as the treatment substrate. The bottles were cleaned, bleached, thoroughly rinsed, and dried in the sun for several days. They were then cut into oyster-shell shaped and sized pieces. The beverage bottles had natural crevasses and concavity like the shell areas preferred by larval oysters [88]. The plastic was then lightly sanded to create microtexture for adherence prior to metamorphosis [89]. This also created initial fragmentation to begin the plastic degradation process in the marine system. Six mesh bags used in oyster restoration, three filled with shell and three filled with PET plastic, were placed in a settling tank, along with numerous shell bags used for restoration, at St. Mary’s College of Maryland on July 13, 2017. Over 3 million pediveliger larvae reared at Horn Point hatchery of University of Maryland were released into the tank to settle on substrate and metaphorize into juvenile oysters. On July 20, 2017, the bags were removed, and the juvenile oysters on substrate were transferred to metal cages. They were then outplanted in a pairwise fashion, one shell cage and one plastic cage, at three locations on the restoration reefs of the St. Mary’s River, Maryland, USA. This work was completed under permit 2012-N-005-M1-R1 issued by the Maryland Department of Natural Resources Fishing and Boating Services to the St. Mary’s River Watershed Association. Shell and plastic substrate cages were placed at least 5m apart on the reef. On May 29, 2018, the cages were collected under permit SCP201826 issued by the Maryland Department of Natural Resources Fishing and Boating Services to the St. Mary’s River Watershed Association. The approximately 10-month-old oysters were transferred to two recirculating tanks at SUNY Cortland, one for shell substrate and one for plastic substrate. Each tank was held at 13ppt, matching the salinity of the St. Mary’s River at the time of collection. The oysters were fed Shellfish Diet 1800 at broodstock conditioning concentrations until they could be sampled for tissue the following week.

### Tissue sampling

Oysters were shucked and sampled one at a time in a single day. After opening the oyster, gill tissue and gonad tissue were carefully excised using a dissecting microscope and immediately flash frozen in liquid nitrogen. The microcentrifuge tubes were then stored in a -80°C freezer. A pipette was used to make a wet mount slide of a small piece of gonad that was then observed with a light microscope to identify the sex and degree of maturity of the oyster. Mature females were identified as having abundant fully round eggs. Mature males were identified as having mobile sperm. Shell length from hinge to apex was measured with calipers. A total of hundred oysters were sampled, fifty growing on shell and fifty growing on plastic.

### RNA extraction and RNA-seq

We extracted RNA from 48 tissue samples: 6 male gill samples from plastic exposure (PE), 6 male gonad samples from PE, 6 male gill samples from shell exposure (SE), 6 male gonad samples from SE, 6 female gill PE, 6 female gonad PE, 6 female gill SE, and 6 female gonad SE. Each gill and gonad sample for a type of exposure came from the same individual oyster. For example, the 6 male gill PE and 6 male gonad PE were excised from the same 6 oysters. Tissue extraction was completed using the RNeasy RNA Mini-Kit with Qiashredder, and the RNA was purified using the RNeasy MinElute Cleanup Kit (Qiagen®, Netherlands). We then isolated the mRNA from the total RNA using the NEBNext Poly(A) mRNA Magnetic Isolation Module (New England BioLabs® Inc., USA). The quality of the mRNA was checked using the Bioanalyzer at Cornell University. We measured the concentration of mRNA using a Qubit ® RNA HS Assay (Invtrogen™, USA) The mRNA library for each of the 48 samples was prepared using the NEBNext Ultra II Directional RNA Library Prep (New England BioLabs® Inc., USA). Each sample had unique oligonucleotides annealed to the mRNA sequences using the NEBNext Multiplex Oligos for Illumina Primer Index kit New England BioLabs® Inc., USA). Each library was then analyzed again for concentration via Qubit® DNA HS Assay and quality via the Bioanalyzer. We then pooled together 12 samples into a single library for sequencing, generating a total of 4 pooled libraries. The samples were sent to Novogene for high-throughput paired-end sequencing on their Illumina NovaSeq 6000 Platform PE150, using 4 lanes with one library per lane. Novogene completed an additional clean-up step to remove primer/adapter contamination prior to sequencing.

### Read alignment to reference transcriptome

Raw reads were cleaned using fastp v0.2.0 [90]. Cleaned reads from male and female oysters raised on shell or plastic were pseudomapped to the genomic coding regions from the *Crassostrea virginica* version 3 genome [91] using kallisto v0.46.0 [92] with the align_and_estimate_abundance.pl script in Trinity 2.12.0. The genomic coding file was annotated with Trinotate v3.2.2 [93] and TransDecoder v5.5.0 [94] by searching the SwissProt database [95] with BLASTx and BLASTp [96].

### Analysis of differential expression

Differentially expressed genes were identified using the edgeR v4.0.2 package [97] implemented in R [98]. Sample libraries were filtered to remove genes that did not have sufficient counts of 10 total reads per gene. Read counts were then normalized, and “tagwise” dispersions (variance in read counts for each contig) were estimated in edgeR before fitting each contig to a GLM log-linear negative binomial model using the prior.count argument. Gill tissue and gonad tissue expression were analyzed separately because the two tissues have distinct functions that require the expression of different complements of genes. Separate analyses allowed for each tissue to be modeled based on the distinct genes represented in the tissue instead of modeling with thousands of additional genes not expressed by the specific tissue. This gave the analysis larger power to detect significant factors impacting expression despite the small number of individual oysters sequenced. Expression patterns were first explored using multidimensional scaling implemented in edgeR and analyzed using a permutational multivariate analysis of variance using the adonis2 function from the vegan v2.6-6.1 package in R [99]. Expression levels were modeled using 3 fixed effects 1) sex (male or female), 2) substrate (shell or plastic), and 3) sex-by-substrate interaction (SE ×  SU), and the significance of each factor was tested using separate likelihood ratio tests. To further test for substrate and sex-by-substrate patterns of differential expression, we completed 6 planned contrasts, comparing 4) females on shell to females on plastic, 5) males on shell to males on plastic, 6) females on shell to males on plastic, 7) males on shell to females on plastic, 8) males on shell to females on shell, and 9) males on plastic to females on plastic for a total of 9 likelihood ratio tests per contig. All contrasts were completed to ensure that candidate genes that were significant for the SE ×  SU term were differentially expressed between groups that had biological significance. The resulting *P*-values from all 9 sets of likelihood ratio test comparisons were corrected for multiple comparisons using the false discovery rate (FDR) [100], and genes with a FDR < 0.05 were considered significantly differentially expressed. Author-generated code for these analyses is available through GitHub at github.com/LEierman/OysterPlasticRNASeq.

### Gene ontology enrichment

GO enrichments were performed using GoSeq v1.34.1[101] with the scripts implemented in Trinotate v3.2.2 and Trinity v2.15.0. The annotation for the genomic coding regions of the genome was separated into two files: genes expressed in the gonads and genes expressed in the gills. The Trinity script run_GOseq.pl included the assigned GO terms, length of each gene, and the list of all genes expressed in the gonads or the gills.

## Results

### Read quality and pseudoMapping

Paired-end sequencing of 48 samples resulted in 2,042,258,491 raw reads with an average of 42,547,051.9 reads per sample. After filtering for quality, samples averaged 39,443,810 reads. Of these, an average of 23,885,676 reads per sample (60.6%) pseudoaligned to the reference RNA sequences from the annotated genome. After removing genes with less than 10 total reads, 62,613 reference genes (94%) were used in analysis of differential gene expression. The average depth of coverage per sample per gene was 379.1 reads. Sequencing reads are available in the Sequence Reach Archive with accession numbers SRR27687858-SRR27687905 associated with bioproject PRJNA1049959.

### Differential expression in gill tissue

**Fig 1:**
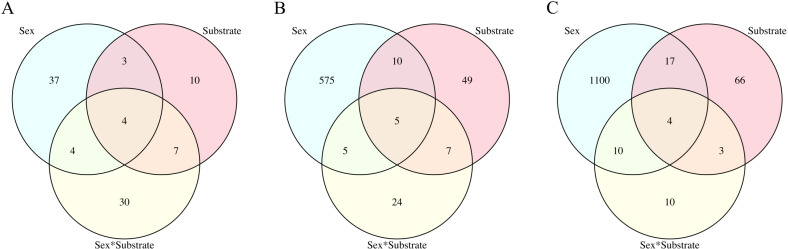
Venn diagrams of differentially expressed genes by model factor in each tissue. Venn diagrams indicating the number of reference genes differentially expressed in response to model factors sex, substrate, and sex-by-substrate for (A) gill tissue, (B) gonad tissue, and (C) gonad tissue after the removal of the P8 male on plastic sample.

**Fig 2:**
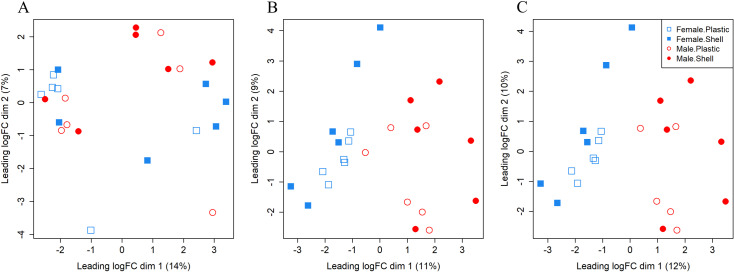
Multidimensional scaling plots of samples for each tissue. Multidimensional scaling plots indicating the clustering of female and male samples on shell and plastic substrate based on patterns of read counts in all expressed genes for (A) gill tissue, (B) gonad tissue, and (C) gonad tissue after the removal of the P8 male on plastic sample.

A total of 41,899 reference genes were expressed across gill tissue samples, with 95 genes (0.22%) differentially expressed (DE) across model factors (Fig 1A, S1 Fig). There was no grouping of samples by sex or by substrate (Fig 2A) with no factor significant in clustering (sex: F = 0.86, df = 1, p = 0.601; substrate: F = 01.12, df = 1, p = 0.279; SE ×  SU: F = 0.85, df = 1, p = 0.572), in keeping with the small percentage of genes with differential expression across model factors. Four of the 95 genes demonstrated significant differential expression in response to all three model factors with the highest expression in female oysters on shell substrate and no gene expression by females on plastic (Fig 1A, S1 Fig). These genes were *multimerin-1-like* (XM_022448393.1), *fibroin heavy chain-like* (XM_022476860.1), uncharacterized LOC111136835 (XR_002639791.1), and *mantle protein-like* (XM_022454702.1).

**Fig 3:**
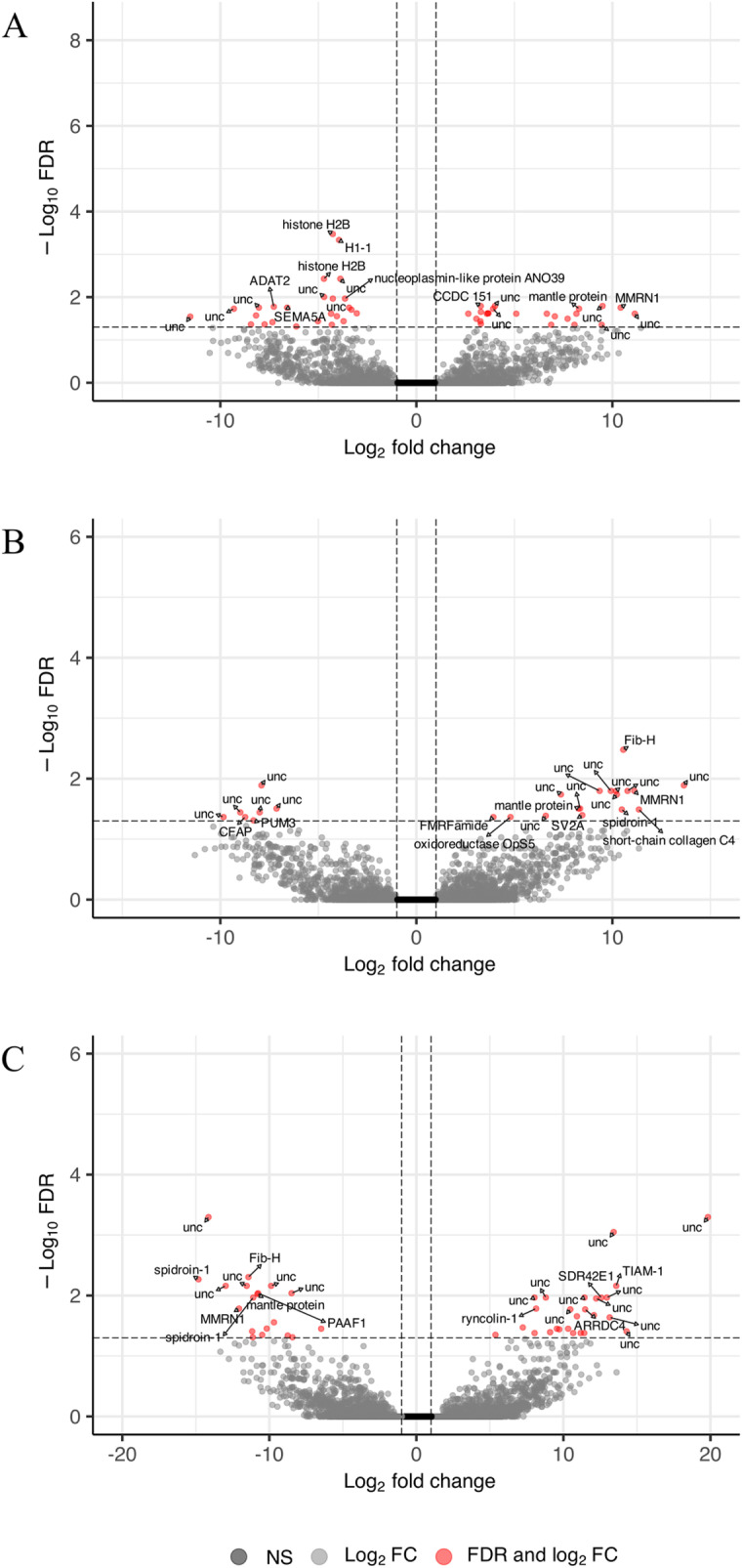
Volcano plots of genes expressed in gill tissue by model factor. Volcano plot log_2_ fold change in expression and false discovery rate of reference genes (n = 41,899) expressed in gill tissue in response to model factors (A) sex, (B) substrate, and (C) sex-by-substrate with differentially expressed genes indicated in red and a subset of genes labeled with gene annotation.

Forty-eight genes were differentially expressed between the sexes (Fig 1A) with 23 more highly expressed in males than females and 25 more highly expressed in females than males (Fig 3A). Of these, 37 genes showed distinct sex-specific patterns unaffected by substrate with 15 more highly expressed in males and 22 more highly expressed in females. Male-specific genes included nine uncharacterized loci, *transcription factor RFX4-like* (XM_022484541.1), *kelch-like protein 2* (XM_022445910.1), *coiled-coil domain-containing protein 151-like* (XM_022469991.1), *papilin-like* (XM_022480399.1), *coiled-coil domain-containing protein 63-like* (XM_022488935.1), and *protein PFC0760c-like* (XM_022464423.1) (Supplementary File 1). Female-specific genes included 10 uncharacterized loci, *tRNA-specific adenosine deaminase 2-like* (XM_022486330.1), *semaphorin-5A-like* (XM_022438096.1), *putative universal stress protein SAS1637* (XM_022475823.1), *histone H2B-like* (XM_022467989.1 and XM_022467992.1), *inactive pancreatic lipase-related protein 1-like* (XM_022489335.1), *vitellogenin-like* (XM_022461020.1), *histone H1-like* (XM_022463009.1), *zinc metalloproteinase dpy-31-like* (XM_022431767.1), *nucleoplasmin-like protein ANO39* (XM_022433937.1 and XM_022433936.1), *alpha-crystallin B chain-like* (XM_022435711.1) (S1 File).

Twenty-four genes were differentially expressed between substrate groups (Fig 1A) with 17 genes more highly expressed by oysters on shell and seven genes more highly expressed by oysters on plastic (Fig 3B, S1 File). Six of the 17 genes that had higher expression on shell and therefore reduced expression in response to plastic were only differentiated by the substrate factor. These were uncharacterized LOC111116296 (XR_002637017.1), uncharacterized protein K04H4.2-like (XM_022471172.1), *short-chain collagen C4-like* (XM_022436894.1), *oxidoreductase OpS5-like* (XM_022465310.1), and *FMRF-amide neuropeptides-like* (XM_022459365.1). Plastic fully suppressed expression in some genes such as uncharacterized protein K04H4.2-like or lowered expression as in uncharacterized locus 111126514 (XM_022471207.1) that also responded to the sex-by-substrate factor (S2 Fig). Of the seven genes more highly expressed in response to plastic, only four responded to the substrate factor. These were three uncharacterized loci and *pumilio homolog 3-like* (XM_022460099.1). Plastic induced activation of genes that were not expressed in female oysters on shell such as *pumilio homolog 3-like* and uncharacterized locus 111122732 (XM_022464651.1), which also responded to the sex-by-substrate factor (S2 Fig).

Forty-five genes were differentially expressed in response to the sex-by-substrate interaction (Figs 1A and 3C). Thirty-eight of these genes demonstrated differential expression in a contrast comparing the difference in expression between males and females on shell to the difference in expression between males and females on plastic (S1 Table). The resulting reaction norms were typical of genotype-by-environment interactions with plastic exposure resulting in the opposite pattern of expression as found in oysters on shell. There was no significant enrichment or depletion by GO term for any group of differentially expressed genes by any model factor.

### Differential expression in gonad tissue

A total of 35,736 reference genes were expressed across gonad tissue samples with 675 genes (1.89%) differentially expressed across model factors (Fig 1B, S2 File). Samples generally separated by sex in multidimensional scaling (Fig 2B), although no factor was significant in clustering (sex: F = 2.29, df = 1, p = 0.069; substrate: F = 0.98, df = 1, p = 0.407; SE ×  SU: F = 0.67, df = 1, p = 0.623). One male sample grown on plastic clustered among the female oysters grown on plastic, referred to as sample P8, resulting in no statistically significant clustering by sex. The females on plastic clustered more closely together on axis two relative to the females on shell that were spread along axis two (Fig 2B), although not statistically significant.

**Fig 4:**
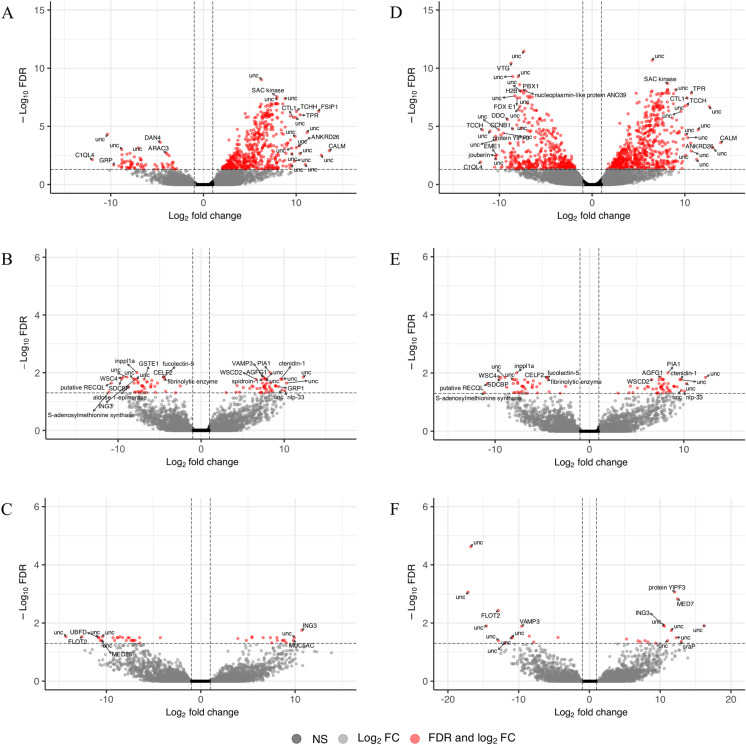
Volcano plots of genes expressed in gonad tissue by model factor. Volcano plot log_2_ fold change in expression and false discovery rate of reference genes expressed in gonad tissue (n = 35,736) in response to model factors (A) sex, (B) substrate, and (C) sex-by-substrate and in gonad tissue after the removal of male on plastic sample P8 (n = 37,676) in response to model factors: (D) sex, (E) substrate, and (F) sex-by-substrate with differentially expressed genes indicated in red and a subset of genes labeled with gene annotation.

**Fig 5:**
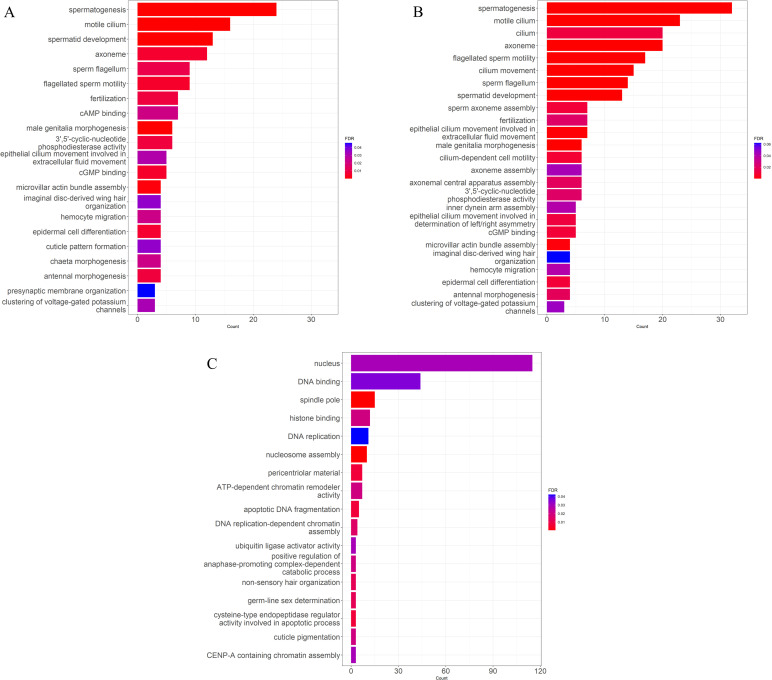
Enriched gene ontology terms of differentially expressed genes in gonadal tissue by sex. Enriched gene ontology terms for genes differentially expressed in gonadal tissues indicating the number of genes and the false discovery rate of each term for (A) males, (B) males after the removal of male on plastic P8, and (C) females after the removal of male on plastic P8.

There were 595 genes differentially expressed in the gonad tissue (Fig 1B) in response to the sex of the oyster with 71 genes more highly expressed in females and 524 genes more highly expressed in males (Fig 4A). The male oyster P8 expressed male-specific genes that were inactive in females, although at the lower range of expression levels (S3 Fig). Similarly, P8 had low levels of expression of genes upregulated in females, although at times slightly higher than other male oysters (S3 Fig). The genes more highly expressed in males were enriched for 21 GO terms primarily related to sperm production (Fig 5A) and were not depleted for any GO terms. Examples of highly expressed genes in males included c*almodulin* (XM_022475832.1), *protein singed-like* (XM_022454659.1 and XM_022447380.1), *spindle assembly checkpoint kinase-like* (XM_022471154.1) and *testis-specific serine/threonine-protein kinase 3-like* (XM_022461085.1). Highly expressed female genes included *vitellogenin-like* (XM_022461020.1), *forkhead box protein L2-like* (XM_022489465.1 and XM_022489697.1), *forkhead box protein E4-like* (XM_022478555.1), *histone H2B-like* (XM_022467992.1), *cell wall protein DAN4-like* (XM_022461132.1), and *phosphoethanolamine N-methyltransferase 3-like* (XM_022481064.1). No GO terms were significantly enriched or depleted in female oysters.

**Fig 6:**
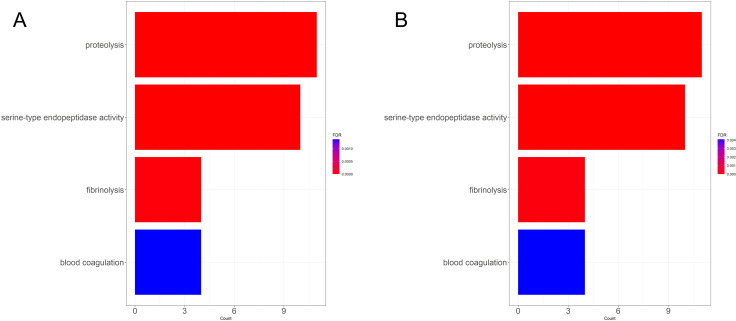
Enriched gene ontology terms of differentially expressed genes in gonadal tissue by plastic. Enriched gene ontology terms for genes differentially expressed in gonadal tissues indicating the number of genes and the false discovery rate of each term for (A) oysters on plastic and (B) oysters on plastic after the removal of male on plastic P8.

Seventy-one genes were differentially expressed in the gonad tissue in response to substrate (Fig 1B). Thirty-three genes had significantly higher expression in oysters grown on plastic and 38 genes had significantly higher expression in oysters grown on shell (Fig 4B). Exposure to plastic elevated expression regardless of sex in genes such as *fibrinolytic enzyme, isozyme C-like* (XM_022465831.1, XM_022466083.1, XM_022466148.1, XM_022471877.1, and XM_022472866.1), *chymotrypsin-like serine proteinase* (XM_022470590.1, XM_022470662.1, XM_022472606.1, and XM_022472607.1) *tetraspanin-18-like* (XM_022465579.1), *methyltransferase-like protein 25* (XM_022476717.1) and *cell wall integrity and stress response component 4-like* (S4 Fig). The genes upregulated in response to plastic were significantly enriched for the processes of proteolysis, serine-type endopeptidase activity, fibrinolysis, and blood coagulation (Fig 6A) and were not depleted for any terms. Exposure to plastic suppressed expression in genes such as *glycine-rich cell wall structural protein 1-like* (XM_022457747.1), *glycine-rich protein 23-like* (XM_022490153.1), *glycine-rich cell wall structural protein-like* (XM_022457679.1, XM_022462208.1), *glycine-rich cell wall structural protein 1.0-like* (XM_022478012.1, XM_022478014.1), *spidroin-1-like* (XM_022477923.1, XM_022477925.1, XM_022476861.1), *glycine, alanine and asparagine-rich protein-like* (XM_022478013.1), *ctenidin 1-like* (XM_022489166.1) (S4 Fig). The genes downregulated in response to plastic were not enriched or depleted for any GO term.

There were 41 genes differentially expressed in the gonad tissue in response to the interaction of sex-by-substrate (Figs 1C and 4C). Several genes demonstrated classic genotype-by-environment interactions with plastic elevating expression in females and reducing expression in males (S5 Fig). From planned contrasts, most genes were upregulated in response to plastic within a sex (i.e., females on plastic compared to females on shell) (S2 Table). They were also upregulated in females, regardless of substrate, compared to males on shell (S2 Table). A notable gene, *vitellogenin*, had higher expression in females on shell, females on plastic, and males on plastic relative to males on shell with no significant difference among the three groups (S5 Fig, S2 Table). There was no significant GO enrichment or depletion for any group of differentially expressed genes within the sex-by-substrate factor.

The uneven distribution of genes differentially expressed by sex led to additional analyses to see how expression patterns changed when P8, the male on plastic grouped with females on plastic in multidimensional scaling (Fig 2B), was removed from the dataset (Fig 2C). Samples were significantly clustered by sex but not by substrate or sex-by-substrate (sex: F = 2.98, df = 1, p = 0.022; substrate: F = 0.65, df = 1, p = 0.609; SE ×  SU: F = 0.50, df = 1, p = 0.776). After removing P8 and then repeating the steps to filter and normalize the libraries, the number of reference genes sufficiently expressed in gonad tissue for analysis increased to 37,676 genes. Of these, 1,210 genes were identified as differentially expressed across model factors (Fig 1C, S3 File).

A total of 1,131 genes were differentially expressed between male and female oysters (Fig 1C). Six hundred eighty-three genes were more highly expressed in males and 448 genes were more highly expressed in females (Fig 4D). The two gonadal analyses overlapped by 585 genes differentially expressed in both. Only 10 genes originally identified as differentially expressed were no longer significant after removing the P8 oyster from analysis. An additional 546 genes were identified as differentially expressed, most with significantly higher expression in females than males. The GO enrichment analysis found similar enrichment in “male” genes as were found in the original analysis (Fig 5B). In females, GO enrichment identified overrepresentation in functions related to the nucleus, DNA replication, mitosis, and meiosis (Fig 5C).

Ninety genes were differentially expressed in response to oyster substrate with 69 of the original 71 genes still differentially expressed after removing P8 from the analyses and 21 more genes differentially expressed (Fig 1C). Forty-eight genes were more highly expressed by oysters on shell, and 42 genes were more highly expressed by oysters on plastic (Fig 4E). The genes more highly expressed in oysters on plastic remained enriched for the processes of proteolysis, serine-type endopeptidase activity, fibrinolysis, and blood coagulation (Fig 6B).

The changes in genes significantly differentially expressed in response to the sex-by-substrate factor were more numerous than substrate alone due to the large change in genes identified as responding to the sex factor. Of the original 41 genes identified, only 14 were still differentially expressed and a different 13 genes were identified for a total of 27 differentially expressed genes (Figs 1C and 4F). Of the 14 genes DE in both analyses, five were characterized: *vesicle-associated membrane protein 3-like* (XM_022485445.1), *inhibitor of growth protein 3-like* (XM_022446094.1), *E3 ubiquitin-protein ligase XIAP-like* (XM_022432213.1), *phosphatidylinositol 3,4,5-trisphosphate 5-phosphatase 2A-like* (XM_022479699.1), and *flotillin-2a-like* (XM_022483964.1). Within sex comparisons found an equal number of genes with elevated expression and decreased expression in response to plastic (S3 Table).

### Tissue comparison

**Fig 7:**
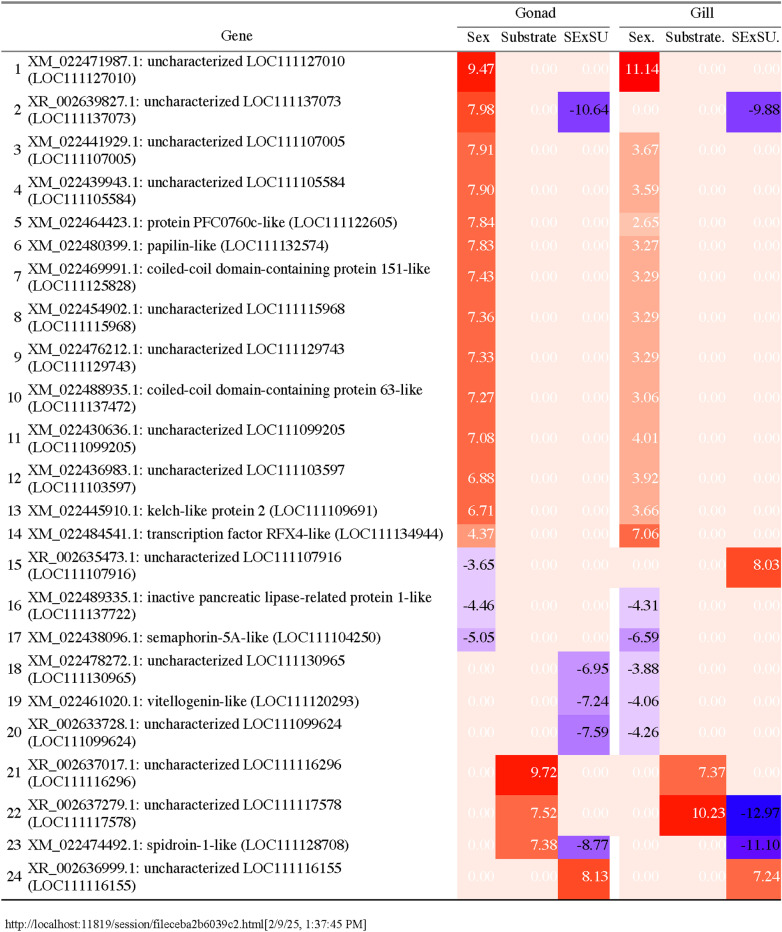
Model results for twenty-four genes differentially expressed in both gill and gonadal tissue. Model results for each of three factors (Sex, Substrate, and Sex-by-substrate) for twenty-four genes differentially expressed in both gill and gonadal tissue. For genes with differential expression for a model factor, the log fold change is provided. The sex factor indicates male expression relative to female ( + male/- female). The substrate factor indicates shell expression relative to plastic ( + shell/- plastic). The sex-by-substrate (SExSU) factor indicates male on shell expression relative to all other groups. Heat map colors are red for positive log-fold changes and blue for negative log-fold changes. Non-significant factors for a gene are indicated as 0 log-fold change.

Gill and gonadal tissue expressed the same 33,098 genes. In comparing the genes differentially expressed in gill and gonadal tissue, only 24 genes were differentially expressed in both tissues. Of these, 15 genes were differentially expressed due to sex in both tissues, two in response to substrate, and three in response to sex-by-substrate. Three genes were differentially expressed by sex in the gill tissue but by the sex-by-substrate interaction term in the gonad tissue. One gene was differentially expressed by the sex-by-substrate term in the gill tissue but by the sex term in the gonadal tissue. All 24 genes showed the same direction of increased expression in both tissues (Fig 7).

## Discussion

First-year oysters exposed to plastic debris throughout their first 10 months demonstrated altered gene expression in both gill and gonadal tissue, with sex-specific patterns identified in both. Gene functions of differentially expressed genes fell into one of three broad functions: (1) gametogenesis, (2) biomineralization and growth, and (3) immune and stress responses. Differential expression by sex in both tissues was primarily in genes involved in the development of sperm and oocytes. Exposure to plastic decreased or terminated the expression of biomineralization genes and increased the expression of immune and stress-related genes in both gill and gonadal tissues. Sex-by-substrate interactions modulated the response of immune and stress-related genes to plastic by sex in gonadal tissue. These findings suggest that exposure to plastic debris may reduce growth, inhibit shell development, and increase stress in oysters as they develop.

Four genes were differentially expressed in gill tissue in response to sex, substrate, and sex-by-substrate factors with no transcription in females exposed to plastic (S1 Fig). *Multimerin-1-like* (XM_022448393.1) is part of the C1q family of proteins involved in innate immunity [102] and had decreased expression in response allograft stress in the pearl oyster [103]. Both *fibroin heavy chain-like* (XM_022476860.1) and *mantle protein-like* (XM_022454702.1) are involved in shell formation [104,105]. Additionally, mantle proteins are involved in the vesicle transport of immune components from the mantle tissue [106]. The suppression of these genes in response to plastic in female gill tissue may result in slower growth and reduced immune function. Protandric oysters begin to develop as females when they reach approximate 60mm in shell length [107]. However, Sorini et al. (2021) found that first-year male and female oysters grown with shell and plastic did not differ significantly in size and were largely below the size threshold to develop as females, despite a sex ratio skewed female for oysters on plastic. This female-bias, despite their small size, may possibly be due to sex-dependent differences in expression of biomineralization genes.

Gill tissue displayed additional sex differences in gene expression. Male oysters had higher expression of genes involved in sperm formation such as *coiled-coil domain-containing protein 151-like* (XM_022469991.1), *coiled-coil domain-containing protein 63-like* (XM_022488935.1), and *transcription factor RFX4-like* (XM_022484541.1) [86,108,109]. Additionally, male gill tissue had higher expression of *papilin-like* (XM_022480399.1), a gene involved in shell formation via the nacre layer [110] and *kelch-like protein 2* (XM_022445910.1), a member of the kelch family that functions in ubiquination and is therefore important in the breakdown of proteins as part of an immune response [111]. Most of these genes were also upregulated in male gonadal tissue (Fig 7). Females had higher expression of genes involved in oocyte development such as *histone H1-like* (XM_022463009.1) and *vitellogenin-like* (XM_022461020.1) [86]. The other genes more highly expressed in female gill tissue are involved in immune and stress responses. Vitellogenin-like genes are up-regulated in response to estrogenic compounds and are indicative of endocrine disruption by BPA and octylphenol [112,113]. Histone H1-like proteins are part of the immune response of invertebrates [114] and are upregulated in clam hemocytes after challenge with *Perkinsus* [115]. Additional upregulated genes included *semaphorin-5A-like* (XM_022438096.1), *putative universal stress protein SAS1637* (XM_022475823.1), *histone H2B-like* (XM_022467989.1, XM_022467992.1), *zinc metalloproteinase dpy-31-like* (XM_022431767.1), *inactive pancreatic lipase-related protein 1-like (*XM_022489335.1), and *alpha crystalline B chain-like* (XM_022435711.1). *Semaphorin-5A-like* is involved in cell proliferation and migration, and it is downregulated in the digestive gland of clams after exposure to progestin [116] and has disrupted expression patterns in mussels after exposure to herbicides [117]. Putative universal stress proteins and *alpha-crystallin B chain-like* are upregulated in snails that are resistant to *Schistoma* infections [118]. *Alpha-crystallin B chain-like* is also responsive to heat shock in *C. gigas* [119]. *Histone H2B-like* has antibacterial properties and is active against *Vibrio* species [120,121]. *Zinc metalloproteinase* and *pancreatic lipase-related protein 1-like* are responsive to metal stress in *C. gigas* [122]. Several of these genes were also upregulated in female gonad tissue (Fig 7). These differences suggest that female oysters might be more responsive to stress from plastic, at least while reproductively active, than are male oysters, similar to the responsiveness of female *M. galloprovincialis* to polyethylene microplastic ingestion [48].

Gill tissue responded to the presence of plastic substrate with the deactivation of genes involved in shell formation and growth and the activation of genes responsive to stress. *Spidroin-1-like* (XM_022476864.1), *synaptic vesicle 2-related protein-like* (XM_022447626.1), uncharacterized *protein K04H4.2-like* (XM_022471172.1), *oxidoreductase OpS5-like* (XM_022465310.1), and *short-chain collagen C4-like* (XM_022436894.1) were more highly expressed by oysters on shell substrate and suppressed by oysters on plastic substrate, along with multimerin-1-like, fibroin heavy chain-like, and mantle protein-like, discussed above. Spidroin genes are involved in biomineralization of the shell [105] and also have a SNP associated with resistance to *Vibrio* infections [123]. *Oxidoreductase OpS5-like* and *short-chain collagen C4-like* are also involved in shell biomineralization [124,125]. *Synaptic vesicle 2-related protein-like* has a growth-related SNP in the Pacific abalone [126]. *Protein K04H4.2* is a secreted protein that binds chitin in *Caenorhabditis elegans* [127] and might therefore play a similar role via shell formation in bivalves. Two identified genes were upregulated in response to plastic: *pumilio homolog 3-like* (XM_022460099.1) and *cilia- and flagella-associated protein 221-like* (XM_022454549.1). *Pumilio homolog-3* interacts with the C-terminus of FMRP and is correlated with cellular stress levels [128]. The cilia- and flagella-associated protein (CFAP) family is involved in the assembly of cilia and flagella and is indicated in both the stress [129–131] and immune responses of bivalves [132]. Combined, the expression patterns of these genes suggest that biomineralization and growth are reduced in response to plastic debris while immune and stress responses are increased.

In the gonadal tissue, male oysters expressed genes required for spermiogenesis. The enriched GO terms, both with and without the male P8 included in the analyses, were predominantly functions such as spermatogenesis, motile cilium, spermatid development, axoneme, sperm flagellum, and flagellated sperm motility. Top genes differentially expressed included *calmodulin* (XM_022475832.1) a protein that is present in high levels in sperm and important for motility [133], *protein singed-like* (XM_022454659.1 and XM_022447380.1), an actin-binding protein that exhibits a large peak in expression at stage 3 of male oyster gonadal development [85], and *testis-specific serine/threonine-protein kinase 3-like* (XM_022461085.1) necessary for sperm development [134]. With oyster P8 included in the analysis, the differentially expressed genes in females were not significantly enriched for any GO terms. However, once P8 was removed from the analysis, the differentially expressed female genes also matched expectations for enriched female gametogenesis GO terms with top functions such as nucleus, DNA binding, spindle pole, histone binding, and DNA replication. These results were similar to the functions such as chromatin condensation, DNA replication, and mitosis and meiosis activities found in previous studies on gametogenesis in oysters [86]. Other enriched GO terms in females included apoptotic DNA fragmentation, ubiquitin ligase activator activity, and cysteine-type endopeptidase regulator activity involved in apoptotic process. Apoptosis functions are common in gonadal development of bivalves, particularly females, as ovarian follicles die and are reabsorbed [86,135,136]. However, increased rates of apoptosis may also be indicative of environmental stress [137]. Top genes differentially expressed included well-known female-related genes such as *vitellogenin-like* (XM_022461020.1), *forkhead box protein L2-like* (XM_022489465.1 and XM_022489697.1), *forkhead box protein E4-like* (XM_022478555.1), and *histone H2B-like* (XM_022467992.1) [85,86].

Interestingly, the gonadal tissue showed responses to substrate that were similar to those observed in the gill tissue, with decreased expression of genes involved in biomineralization and increased expression of immunity and stress-related genes. Down-regulated biomineralization genes included *ctenidin-1-like* (XM_022489166.1), *glycine-rich cell wall structural protein 1-like* (XM_022457747.1), *glycine-rich protein 23-like* (XM_022490153.1), *glycine-rich cell wall structural protein-like* (XM_022457679.1, XM_022462208.1), *glycine-rich cell wall structural protein 1.0-like* (XM_022478012.1, XM_022478014.1), *spidroin-1-like* (XM_022477923.1, XM_022477925.1, XM_022476861.1), and *glycine, alanine and asparagine-rich protein-like* (XM_022478013.1) [104,105,138]. Also down-regulated were *neurogenic locus Notch protein-like* (XM_022470729.1), which is an immune-related molecule involved in neuroendocrine modulation of hemocytes [139], *L-rhamnose-binding lectin CSL3-like* (XM_022488355.1) from a family of genes involved in inflammatory responses to bacterial infections [140], and *caveolin-1-like* (XM_022453101.1), a gene downregulated in male mussels exposed to the flame retardant tetrabromobisphenol A [141]. Downregulation of biomineralization, immune and stress-related genes suggest that plastic exposure may reduce growth and make oysters more susceptible to some types of infections.

The genes with significantly increased expression in response to plastic were enriched for three major GO terms: proteolysis, serine-type endopeptidase activity, and fibrinolysis. Proteolysis is a critical part of an organism’s stress response, removing damaged and malfunctioning proteins [142–144], as seen in bivalves responding to lowered pH, trace metal exposure, and thermal stress [145,146]. Serine-type endopeptidase activity also indicates the breakdown of proteins through the hydrolysis of peptide bonds. Similarly, fibrinolysis breaks down fibrin as a symptom of infection as seen in response to *Perkinsus marinus* [147], or fibrinolysis can participate in the immune signaling pathway [148,149]. Many of the upregulated genes are responsive to either pathogens or stress in various bivalves. *Fibrinolytic enzyme, isozyme C-like* (XM_022465831.1, XM_022466083.1, XM_022466148.1, XM_022471877.1, and XM_022472866.1) and *chymotrypsin-like serine proteinase* (XM_022470590.1, XM_022470662.1, XM_022472606.1, and XM_022472607.1) were significantly upregulated in eastern oysters exposed to crude oil [150]. Chymotrypsin-like serine proteinases are also important to the immune function of shrimp [151]. *Tetraspanin-18-like* (XM_022465579.1) is from a family of tetraspanins involved in the innate immune response, functioning in pattern recognition of pathogens [152–155] and upregulated in response to *Perkinsus* infections [156]. *Methyltransferase-like protein 25* (XM_022476717.1) is involved in the epitranscriptomic regulation of biological processes [157]. It is upregulated in clams exposed to BDE-47, a type of flame retardant found in various materials including plastic [158], and it is also upregulated in oyster larvae exposed to conditions consistent with ocean acidification [159].

Additional genes showed patterns of expression in response to plastic that differed by sex. These genes were again indicated as functioning in response to stress or as an immune response to a pathogen. Five genes maintained the same pattern regardless of the inclusion of P8 in the analysis. These genes were *vesicle-associated membrane protein 3-like* (XM_022485445.1), *inhibitor of growth protein 3-like* (XM_022446094.1), *E3 ubiquitin-protein ligase XIAP-like (*XM_022432213.1), *phosphatidylinositol 3,4,5-trisphosphate 5-phosphatase 2A-like* (XM_022479699.1), and *flotillin-2a-like* (XM_022483964.1). *Inhibitor of growth protein 3-like* is a tumor suppressor protein that in humans prevents the activation of the innate immune system [160]. It is expressed by clams infected with a quahog parasite but downregulated at cooler temperatures [161] and upregulated in clams after lead exposure [162]. *E3 ubiquitin-protein ligase XIAP-like* regulates a cell death pathway by binding to caspase 3 to inhibit apoptosis [163] and was significantly upregulated in multiple studies investigating the impacts of pathogen and environmental stress on molluscs, including pathogen associated molecular patterns from bacteria in snails [164], Roseovarius oyster disease in oysters [165], and air exposure in clams [166]. *Phosphatidylinositol 3,4,5-trisphosphate 5-phosphatase 2A-like* is part of the P13K-Akt signaling pathway and is upregulated in oyster larvae challenged with *Vibrio corallilytics* [167,168]. Flotillin proteins are found in the hemocytes of bivalves, specifically granulocytes involved in immune responses [169] and hyalinocytes involved in clotting [170]. Additional genes with sex-by-substrate patterns include *fucolectin-5-like* (XM_022452411.1) as well as *vitellogenin-like* (XM_022461020.1) and *spidroin-1-like* (XM_022474492.1), both described above. *Fucolectin-5-like* is a member of the fucose-binding lectin group that is involved in innate immunity, including activation of the complement system and adaptive immune response [167,168]. Additionally, fucolectins are uniquely expressed in the gonads of oysters, indicating a potential function in reproductive development [171]. The patterns of expression for these various genes are not easily interpreted given the modified response to substrate by sex. However, their roles in immunity and stress suggest that plastic is having a complex impact on oyster immunity and stress.

The intermediate expression patterns of the male oyster on plastic - P8 - leads to additional hypotheses. This oyster expressed “male” genes in its gonad at levels similar to other male oysters, often on the lower end of the range but still higher than the expression observed in females (S3 Fig). Similarly, expression levels of “female” genes identified with P8 in the analysis were often higher for P8 than other males but below the expression level of females (S3 Fig). Furthermore, we reconfirmed the identification of P8 as a male oyster through photographic records of gonadal tissue taken via microscope and data collection on shell length. We hypothesize that P8 may be an example of a protandric oyster responding to endocrine disrupting chemicals from plastic without triggering a change in gamete development from sperm to egg. The clustering via multidimensional scaling across the expression of all analyzed genes (Figs 1B and 1C) showed females on plastic tightly clustered and females on shell more spread along axis 2. It is possible that oysters with the female genotype have higher variance in their expression but that protandric oysters require a particular baseline of expression across specific genes to be met before developing eggs. The male P8 may have fallen below this hypothetical threshold for specific genes but displayed more “feminized” expression relative to other males in response to chemicals leached from the plastic debris.

Some observed expression patterns, particularly in the gill tissue, were more consistent with patterns previously seen in mantle tissue [105]. Given the small size of the 10-month-old oysters used in the study, this may indicate that some mantle tissue may have been attached to the dissected gill tissue, despite the care taken to prevent this. However, it is unlikely that such contamination would have coincided with male/female sampling or with shell/plastic sampling. While a given gene of interest in an individual oyster may have been influenced by a contaminant piece of tissue, the larger patterns of the impact of plastic substrate on differential expression would remain, as seen by the complete termination of transcription within groups of oysters such as females on plastic (S1, S2, and S4 Figs), or the upregulation of expression in oysters on plastic (S2 and S4 Figs). It is also possible that the hemolymph present across all tissues, including gill and gonad, also impacted expression, particularly of immune and stress related genes [86]. The distinct expression of gametogenesis genes in gonadal tissue indicates the large presence of gonad tissue. However, some of the genes that responded to substrate in the gonad are involved in biomineralization of the shell and therefore growth. It is less likely that mantle tissue contamination occurred based on how the gonad was dissected during tissue sampling, but not impossible. Alternatively, signaling may occur across tissues in the oyster during gametogenesis as the energy budget shifts from growth in the spring to reproduction in the summer [172,173] such that the gonad signals the extent of expression of biomineralization genes. The deactivation of these genes in the gonads exposed to plastic could therefore indicate that the additional stress of plastic pollution required a larger change in the energy budget and reduced growth or that endocrine-disrupting chemicals caused an increase in the energy needs of the gonad during gametogenesis.

We conclude that plastic debris represents a complex challenge to eastern oysters, impacting the expression of genes necessary for biomineralization and growth, gametogenesis, and immune- and stress responses. The numerous functions impacted by the observed differential expression corroborates existing studies on the numerous physiological impacts observed in response to plastic. Gene expression in response to plastic was altered by sex, suggesting that females may be more susceptible to stress caused by plastic pollution similar to mussels responding to microplastic ingestion [48] or have larger responses that successfully mitigate large impacts as indicated by the higher survival rate of female oysters exposed to BPA [60]. Furthermore, while some immune and stress-related genes were upregulated in response to plastic, others were suppressed, such as spidroin, a gene implicated in immune responses to *Vibrio* infections. The “plastisphere” of plastic pollution is rich with *Vibrio* species including pathogens, raising the question of whether some immune responses to plastic are initiated by bacteria growing on the plastic and whether plastic may inhibit pathogen-specific immune responses while initiating the infection. Additionally, the reduced expression of biomineralization genes in response to plastic suggest that the energy budget of oysters may be impacted by plastic pollution, either through reduced total energy, reduced digestion and nutrient absorption, or a shift in the budget to immune and stress responses or gonadal development. As a result, plastic may hinder growth and reproductive output while increasing stress in a keystone species already at a historically low population size.

## Supporting information

S1 File GLM log-linear negative binomial model results for gill tissue for 41,899 reference genes. For genes with differential expression for a model factor, the log fold change is provided. The sex factor indicates male expression relative to female ( + male/- female). The substrate factor indicates shell expression relative to plastic ( + shell/- plastic). The sex-by-substrate (SExSU) factor indicates male on shell expression relative to all other groups.
(XLSX)

S2 File GLM log-linear negative binomial model results for gonad tissue for 35,736 reference genes. For genes with differential expression for a model factor, the log fold change is provided. The sex factor indicates male expression relative to female ( + male/- female). The substrate factor indicates shell expression relative to plastic ( + shell/- plastic). The sex-by-substrate (SExSU) factor indicates male on shell expression relative to all other groups.
(XLSX)

S3 File GLM log-linear negative binomial model results for gonad tissue after the removal of the male on plastic (P8) oysters for 37,676 reference genes. For genes with differential expression for a model factor, the log fold change is provided. The sex factor indicates male expression relative to female ( + male/- female). The substrate factor indicates shell expression relative to plastic ( + shell/- plastic). The sex-by-substrate (SExSU) factor indicates male on shell expression relative to all other groups.
(XLSX)

S1 Table Log-fold change between two groups of oysters from planned contrasts for genes differentially expressed in response to the sex-by-substrate factor in gill tissue. Positive log-fold changes are more highly expressed in the first group listed and negative log-fold changes are more highly expressed in the sex group listed. Non-significant comparisons are indicated as 0 log-fold change.
(PDF)

S2 Table Log-fold change between two groups of oysters from planned contrasts for genes differentially expressed in response to the sex-by-substrate factor in gonad tissue. Positive log-fold changes are more highly expressed in the first group listed and negative log-fold changes are more highly expressed in the sex group listed. Non-significant comparisons are indicated as 0 log-fold change.
(PDF)

S3 Table Log-fold change between two groups of oysters from planned contrasts for genes differentially expressed in response to the sex-by-substrate factor in gonad tissue after the male oyster on plastic P8 was removed from the analysis. Positive log-fold changes are more highly expressed in the first group listed and negative log-fold changes are more highly expressed in the sex group listed. Non-significant comparisons are indicated as 0 log-fold change.
(PDF)

S1 Fig Norm of reaction for the four genes differentially expressed in gill tissue in response to all three model factors. Norm of reaction for the four genes differentially expressed in gill tissue in response to all three model factors: sex, substrate, and sex-by-substrate (A) XM_022448393.1: *Multimerin-1-like* (LOC111111409), transcript variant X2, (B) XM_022476860.1: *Fibroin heavy chain-like* (LOC111130129) (C) XR_002639791.1: uncharacterized (LOC111136835) (D) XM_022454702.1: *mantle protein-like* (LOC111115840). Small points are individual read counts and large points are mean read counts with 95% confidence interval for each group of oysters with lines connecting means between sexes of the same substrate.
(TIF)

S2 Fig Norm of reaction for four representative genes differentially expressed in gill tissue in response to substrate. Norm of reaction for four representative genes differentially expressed in gill tissue in response to substrate with (A) and (B) representing genes more highly expressed on shell and (C) and (D) representing genes more highly expressed on plastic: (A) XM_022471172.1: uncharacterized protein K04H4.2-like (LOC111126488), (B) XM_022471207.1: uncharacterized (LOC111126514), (C) XM_022460099.1: *pumilio homolog 3-like* (LOC111119682), transcript variant X3, (D) XR_002637717.1: uncharacterized (LOC111120970). Small points are individual read counts and large points are mean read counts with 95% confidence interval for each group of oysters with lines connecting means between sexes of the same substrate.
(TIF)

S3 Fig Norm of reaction for four representative genes differentially expressed in gonad tissue in response to sex. Norm of reaction for four representative genes differentially expressed in gonad tissue in response to sex with (A) and (B) representing genes more highly expressed in males regardless of substrate and (C) and (D) representing genes more highly expressed in females regardless of substrate: (A) XM_022471154.1: *spindle assembly checkpoint kinase-like* (LOC111126472), (B) XM_022461657.1: *testis-specific serine/threonine-protein kinase 1-like* (LOC111120728), (C) XM_022461132.1: *cell wall protein DAN4-like* (LOC111120418), (D) XM_022481064.1: *phosphoethanolamine N-methyltransferase 3-like* (LOC111133025), transcript variant X3. Small points are individual read counts and large points are mean read counts with 95% confidence interval for each group of oysters with lines connecting means between sexes of the same substrate.
(TIF)

S4 Fig Norm of reaction for four representative genes differentially expressed in gonad tissue in response to substrate. Norm of reaction for four representative genes differentially expressed in gonad tissue in response to substrate with (A) representing genes more highly expressed on plastic and (B) representing genes more highly expressed on shell: (A) XM_022457216.1: *cell wall integrity and stress response component 4-like* (LOC111117955), transcript variant X2 and (B) XM_022489166.1: *ctenidin-1-like* (LOC111137625). Small points are individual read counts and large points are mean read counts with 95% confidence interval for each group of oysters with lines connecting means between sexes of the same substrate.
(TIFF)

S5 Fig Norm of reaction for five representative genes differentially expressed in gonad tissue in response to sex-by-substrate. Norm of reaction for five representative genes differentially expressed in gonad tissue in response to sex-by-substrate: (A)XM_022435151.1: *CUGBP Elav-like family member 2* (LOC111102426), transcript variant X20, (B) XM_022446094.1: *inhibitor of growth protein 3-like* (LOC111109845), transcript variant X1, (C) XM_022458260.1: *syntenin-1-like* (LOC111118686), transcript variant X1, (D) XR_002635236.1: uncharacterized (LOC111106591), (E) XM_022458233.1: uncharacterized (LOC111118638), and (F) XM_022461020.1: *vitellogenin-like* (LOC111120293). Small points are individual read counts and large points are mean read counts with 95% confidence interval for each group of oysters with lines connecting means between sexes of the same substrate.
(TIF)
