# Supplementary material for: Exposure to plastic debris alters expression of biomineralization, immune, and stress-related genes in the eastern oyster (Crassostrea virginica)
Source: PLoS One. 2025 Apr 29;20(4):e0319165. doi: 10.1371/journal.pone.0319165 (PMC12040131; doi:10.1371/journal.pone.0319165)
Supplement: S1 Table — Positive log-fold changes are more highly expressed in the first group listed and negative log-fold changes are more highly expressed in the sex group listed. Non-significant comparisons are indicated as 0 log-fold change. (PDF) [file pone.0319165.s004.pdf]

| | Gene | Male.Plastic.vs.Female.Shell | Male.Shell.vs.Female.Plastic | Female.Shell.vs.Female.Plastic | Male.Shell.vs.Male.Plastic | Male.Plastic.vs.Female.Plastic | Male.Shell.vs.Female.Shell | Male.Shell.Female.Shell.vs.Male.Plastic.Female.Plastic |
|---|---|---|---|---|---|---|---|---|
| 1 | XM_022431879.1: proteasomal ATPase-associated factor 1-like (LOC111100220), transcript variant X1, mRNA | 0.00 | -7.34 | 0.00 | -8.63 | 0.00 | -9.47 | -10.77 |
| 2 | XM_022448393.1: multimerin-1-like (LOC111111409), transcript variant X2, mRNA | 0.00 | 9.48 | 11.12 | 0.00 | 10.42 | 0.00 | -12.06 |
| 3 | XM_022464651.1: uncharacterized LOC111122732 (LOC111122732), transcript variant X5, mRNA | 7.37 | 0.00 | -7.90 | 0.00 | 0.00 | 8.27 | 8.80 |
| 4 | XR_002634302.1: uncharacterized LOC111101513 (LOC111101513), ncRNA | 0.00 | -7.55 | 0.00 | -9.08 | 0.00 | -8.15 | -9.68 |
| 5 | XR_002634549.1: uncharacterized LOC111102745 (LOC111102745), transcript variant X2, ncRNA | -9.89 | 0.00 | 0.00 | 12.01 | -9.31 | 0.00 | 11.43 |
| 6 | XR_002639827.1: uncharacterized LOC111137073 (LOC111137073), ncRNA | 0.00 | -6.48 | 0.00 | -7.67 | 0.00 | -8.69 | -9.88 |
| 7 | XM_022454549.1: cilia- and flagella-associated protein 221-like (LOC111115715), transcript variant X2, mRNA | 0.00 | 0.00 | -8.74 | 0.00 | 0.00 | 9.28 | 10.32 |
| 8 | XM_022454702.1: mantle protein-like (LOC111115840), mRNA | 0.00 | 0.00 | 8.26 | 0.00 | 8.30 | 0.00 | -10.80 |
| 9 | XM_022461715.1: uncharacterized LOC111120759 (LOC111120759), transcript variant X13, mRNA | 0.00 | 0.00 | -7.99 | 0.00 | 0.00 | 7.95 | 13.42 |
| 10 | XM_022476860.1: fibroin heavy chain-like (LOC111130129), mRNA | 0.00 | 0.00 | 10.55 | 0.00 | 6.88 | 0.00 | -11.42 |
| 11 | XR_002639791.1: uncharacterized LOC111136835 (LOC111136835), ncRNA | 0.00 | 0.00 | 9.34 | 0.00 | 8.16 | 0.00 | -14.14 |
| 12 | XM_022431118.1: complement C1q-like protein 4 (LOC111099707), mRNA | 0.00 | 0.00 | 0.00 | 0.00 | 0.00 | 7.79 | 8.04 |
| 13 | XM_022433713.1: uncharacterized LOC111101274 (LOC111101274), transcript variant X3, | 0.00 | 0.00 | 0.00 | 0.00 | 0.00 | -7.79 | -11.54 |

| | | | | | | | | |
|---|---|---|---|---|---|---|---|---|
| | mRNA | | | | | | | |
| 14 | XM_022434093.1: uncharacterized LOC111101556 (LOC111101556), mRNA | 0.00 | 0.00 | 0.00 | 0.00 | -7.74 | 0.00 | 10.47 |
| 15 | XM_022443542.1: uncharacterized LOC111108048 (LOC111108048), transcript variant X2, mRNA | 0.00 | 0.00 | 0.00 | 0.00 | 0.00 | 9.97 | 10.66 |
| 16 | XM_022455213.1: endothelin-converting enzyme 1-like (LOC111116230), mRNA | 0.00 | 0.00 | 0.00 | 0.00 | 5.10 | 0.00 | -6.47 |
| 17 | XM_022471207.1: uncharacterized LOC111126514 (LOC111126514), transcript variant X2, mRNA | 0.00 | 0.00 | 10.77 | 0.00 | 0.00 | 0.00 | -10.49 |
| 18 | XM_022476864.1: spidroin-1-like (LOC111130132), mRNA | 0.00 | 0.00 | 10.48 | 0.00 | 0.00 | 0.00 | -14.81 |
| 19 | XR_002633942.1: uncharacterized LOC111100211 (LOC111100211), ncRNA | 0.00 | 0.00 | 10.18 | 0.00 | 0.00 | 0.00 | -10.18 |
| 20 | XR_002635820.1: uncharacterized LOC111110526 (LOC111110526), transcript variant X2, ncRNA | 0.00 | 0.00 | 0.00 | 0.00 | -8.18 | 0.00 | 9.56 |
| 21 | XR_002637279.1: uncharacterized LOC111117578 (LOC111117578), ncRNA | 0.00 | 0.00 | 10.23 | 0.00 | 0.00 | 0.00 | -12.96 |
| 22 | XR_002639231.1: uncharacterized LOC111133375 (LOC111133375), ncRNA | 0.00 | 0.00 | 0.00 | 0.00 | 0.00 | 8.67 | 12.23 |
| 23 | XM_022431999.1: T-lymphoma invasion and metastasis-inducing protein 1-like (LOC111100292), transcript variant X1, mRNA | 0.00 | 0.00 | 0.00 | 0.00 | 0.00 | 0.00 | 13.59 |
| 24 | XM_022434818.1: putative nuclease HARBI1 (LOC111102167), mRNA | 0.00 | 0.00 | 0.00 | 0.00 | 0.00 | 0.00 | 9.72 |
| 25 | XM_022437938.1: outer dense fiber protein 3-B-like (LOC111104143), transcript variant X2, mRNA | 0.00 | 0.00 | 0.00 | 0.00 | 0.00 | 0.00 | 10.92 |
| 26 | XM_022447058.1: uncharacterized LOC111110518 (LOC111110518), transcript variant X2, | 0.00 | 0.00 | 0.00 | 0.00 | 0.00 | 0.00 | -8.49 |

| | | | | | | | | |
|---|---|---|---|---|---|---|---|---|
| | mRNA | | | | | | | |
| 27 | XM_022448642.1: uncharacterized LOC111111582 (LOC111111582), transcript variant X5, mRNA | 0.00 | 0.00 | 0.00 | 0.00 | 0.00 | 0.00 | 19.83 |
| 28 | XM_022466821.1: arrestin domain-containing protein 4-like (LOC111123995), transcript variant X2, mRNA | 0.00 | 0.00 | 0.00 | 0.00 | 0.00 | 0.00 | 11.48 |
| 29 | XM_022468322.1: adrenocorticotropic hormone receptor-like (LOC111124956), transcript variant X2, mRNA | 0.00 | 0.00 | 0.00 | 0.00 | 0.00 | 0.00 | 9.10 |
| 30 | XM_022474492.1: spidroin-1-like (LOC111128708), mRNA | 0.00 | 0.00 | 0.00 | 0.00 | 0.00 | 0.00 | -11.10 |
| 31 | XM_022476017.1: microfibril-associated glycoprotein 4-like (LOC111129564), mRNA | 0.00 | 0.00 | 0.00 | 0.00 | 0.00 | 0.00 | 5.38 |
| 32 | XM_022484473.1: ryncolin-1-like (LOC111134917), mRNA | 0.00 | 0.00 | 0.00 | 0.00 | 0.00 | 0.00 | 8.15 |
| 33 | XM_022485575.1: zinc finger protein ZFPM1-like (LOC111135471), transcript variant X3, mRNA | 0.00 | 0.00 | 0.00 | 0.00 | 0.00 | 0.00 | 11.41 |
| 34 | XR_002634482.1: uncharacterized LOC111102424 (LOC111102424), transcript variant X2, ncRNA | 0.00 | 0.00 | 0.00 | 0.00 | 0.00 | 0.00 | -11.17 |
| 35 | XR_002635473.1: uncharacterized LOC111107916 (LOC111107916), ncRNA | 0.00 | 0.00 | 0.00 | 0.00 | 0.00 | 0.00 | 8.04 |
| 36 | XR_002635855.1: uncharacterized LOC111110754 (LOC111110754), ncRNA | 0.00 | 0.00 | 0.00 | 0.00 | 0.00 | 0.00 | 11.18 |
| 37 | XR_002636999.1: uncharacterized LOC111116155 (LOC111116155), ncRNA | 0.00 | 0.00 | 0.00 | 0.00 | 0.00 | 0.00 | 7.24 |
| 38 | XM_022443971.1: uncharacterized LOC111108228 (LOC111108228), transcript variant X1, mRNA | 0.00 | 0.00 | 0.00 | 0.00 | 0.00 | 0.00 | 14.02 |
| 39 | XM_022483959.1: leupaxin-like (LOC111134686), transcript variant X4, mRNA | 0.00 | 0.00 | 0.00 | 0.00 | 0.00 | -7.58 | 0.00 |

| | | | | | | | | |
|---|---|---|---|---|---|---|---|---|
| 40 | XM_022444218.1: echinoidin-like (LOC111108378), mRNA | 0.00 | 0.00 | 0.00 | 0.00 | 0.00 | 0.00 | 0.00 |
| 41 | XM_022449228.1: uncharacterized LOC111111994 (LOC111111994), transcript variant X1, mRNA | 0.00 | 0.00 | 0.00 | 0.00 | 0.00 | 0.00 | 0.00 |
| 42 | XM_022454391.1: uncharacterized LOC111115599 (LOC111115599), transcript variant X3, mRNA | 0.00 | 0.00 | 0.00 | 0.00 | 0.00 | 0.00 | 0.00 |
| 43 | XM_022461989.1: short-chain dehydrogenase/reductase family 42E member 1-like (LOC111120933), transcript variant X3, mRNA | 0.00 | 0.00 | 0.00 | 0.00 | 0.00 | 0.00 | 0.00 |
| 44 | XM_022468895.1: uncharacterized LOC111125286 (LOC111125286), transcript variant X1, mRNA | 0.00 | 0.00 | 0.00 | 0.00 | 0.00 | 0.00 | 0.00 |
| 45 | XM_022469192.1: protocadherin gamma-B4-like (LOC111125407), transcript variant X7, mRNA | 0.00 | 0.00 | 0.00 | 0.00 | 0.00 | 0.00 | 0.00 |
