# Supplementary material for: Exposure to plastic debris alters expression of biomineralization, immune, and stress-related genes in the eastern oyster (Crassostrea virginica)
Source: PLoS One. 2025 Apr 29;20(4):e0319165. doi: 10.1371/journal.pone.0319165 (PMC12040131; doi:10.1371/journal.pone.0319165)
Supplement: S2 Table — Positive log-fold changes are more highly expressed in the first group listed and negative log-fold changes are more highly expressed in the sex group listed. Non-significant comparisons are indicated as 0 log-fold change. (PDF) [file pone.0319165.s005.pdf]

	Gene	Male.Plastic.vs.Female.Shell	Male.Shell.vs.Female.Plastic	Female.Shell.vs.Female.Plastic	Male.Shell.vs.Male.Plastic	Male.Plastic.vs.Female.Plastic	Male.Shell.vs.Female.Shell
1	XR_002633972.1: uncharacterized LOC111100276 (LOC111100276), ncRNA	0.00	0.00	8.19	-8.25	8.25	-8.19
2	XM_022432213.1: E3 ubiquitin-protein ligase XIAP-like (LOC111100396), transcript variant X2, mRNA	6.18	0.00	-6.94	0.00	0.00	7.96
3	XM_022432850.1: lectin-like (LOC111100754), mRNA	0.00	-7.67	0.00	-6.78	0.00	-7.99
4	XM_022439393.1: uncharacterized LOC111105230 (LOC111105230), transcript variant X1, mRNA	0.00	-6.65	0.00	-8.91	0.00	-8.56
5	XM_022442718.1: uncharacterized LOC111107494 (LOC111107494), mRNA	0.00	-9.16	0.00	-7.70	0.00	-10.32
6	XM_022445765.1: uncharacterized LOC111109585 (LOC111109585), transcript variant X3, mRNA	5.62	0.00	-6.00	0.00	0.00	7.00
7	XM_022456447.1: solute carrier organic anion transporter family member 4A1-like (LOC111116607), transcript variant X13, mRNA	0.00	-7.76	0.00	-8.60	0.00	-8.28
8	XM_022458973.1: ubiquitin domain-containing protein UBFD1-like (LOC111119116), transcript variant X2, mRNA	0.00	-9.97	0.00	-10.87	0.00	-9.94
9	XM_022461020.1: vitellogenin-like (LOC111120293), mRNA	0.00	-9.03	0.00	-7.08	0.00	-9.18

10	XM_022463278.1: eomesodermin-like (LOC111121836), mRNA	0.00	-6.33	0.00	-5.40	0.00	-7.39
11	XM_022463516.1: serine/threonine-protein kinase mos-like (LOC111121998), mRNA	0.00	-8.10	0.00	-6.59	0.00	-8.92
12	XM_022463555.1: cell division cycle protein 20 homolog (LOC111122028), mRNA	0.00	-7.98	0.00	-5.70	0.00	-8.77
13	XM_022465075.1: coiled-coil domain-containing protein 158-like (LOC111122998), transcript variant X7, mRNA	0.00	-8.09	0.00	-6.89	0.00	-8.92
14	XM_022466512.1: la-related protein 4-like (LOC111123869), transcript variant X4, mRNA	0.00	-5.26	0.00	-6.60	0.00	-6.56
15	XM_022474416.1: uncharacterized LOC111128661 (LOC111128661), mRNA	0.00	-8.21	0.00	-6.88	0.00	-8.55
16	XM_022477709.1: protein pygopus-like (LOC111130559), transcript variant X1, mRNA	6.17	0.00	-6.97	0.00	0.00	7.86
17	XM_022478272.1: uncharacterized LOC111130965 (LOC111130965), transcript variant X1, mRNA	0.00	-8.44	0.00	-6.70	0.00	-8.69
18	XM_022479699.1: phosphatidylinositol 3,4,5-trisphosphate 5-phosphatase 2A-like (LOC111132057), transcript variant X4, mRNA	7.87	0.00	-7.69	0.00	0.00	8.25
	XM_022489306.1:						

19 uncharacterized LOC111137705 (LOC111137705), transcript variant X2, mRNA	0.00	-4.89	0.00	-6.38	0.00	-6.79
20 XR_002633728.1: uncharacterized LOC111099624 (LOC111099624), ncRNA	0.00	-8.34	0.00	-7.20	0.00	-8.73
21 XR_002638311.1: uncharacterized LOC111125106 (LOC111125106), ncRNA	0.00	-12.20	0.00	-10.04	0.00	-12.58
22 XM_022446169.1: uncharacterized LOC111109903 (LOC111109903), transcript variant X1, mRNA	-6.29	0.00	0.00	7.98	-8.24	0.00
23 XM_022452411.1: fucolectin-5-like (LOC111114123), partial mRNA	0.00	-2.90	-4.46	0.00	-3.26	0.00
24 XM_022455267.1: phytochrome-interacting ankyrin-repeat protein 1-like (LOC111116050), transcript variant X4, mRNA	0.00	7.30	8.27	0.00	7.58	0.00
25 XM_022485445.1: vesicle-associated membrane protein 3-like (LOC111135408), transcript variant X1, mRNA	0.00	7.29	7.71	0.00	8.89	0.00
26 XR_002635236.1: uncharacterized LOC111106591 (LOC111106591), ncRNA	-9.70	0.00	0.00	8.25	-10.35	0.00
27 XM_022442508.1: G2 and S phase-expressed protein 1-like (LOC111107342), transcript variant X4, mRNA	0.00	0.00	-6.87	0.00	0.00	5.33
XM_022446094.1: inhibitor of growth protein 3-like						

28	(LOC111109845), transcript variant X1, mRNA	0.00	0.00	-7.91	0.00	0.00	6.24
29	XM_022474492.1: spidroin-1-like (LOC111128708), mRNA	0.00	0.00	7.38	0.00	0.00	-6.03
30	XM_022483964.1: flotillin-2a-like (LOC111134688), transcript variant X1, mRNA	0.00	0.00	0.00	-8.20	0.00	-7.88
31	XM_022442828.1: uncharacterized LOC111107567 (LOC111107567), transcript variant X1, mRNA	0.00	0.00	12.27	0.00	8.58	0.00
32	XR_002638212.1: uncharacterized LOC111124520 (LOC111124520), ncRNA	-6.28	0.00	0.00	0.00	-8.81	0.00
33	XM_022436973.1: E3 ubiquitin-protein ligase rnf213-alpha-like (LOC111103595), mRNA	0.00	0.00	0.00	0.00	0.00	3.28
34	XM_022437610.1: E3 ubiquitin-protein ligase rnf213-alpha-like (LOC111103955), mRNA	0.00	0.00	0.00	0.00	0.00	2.88
35	XM_022449961.1: uncharacterized LOC111112448 (LOC111112448), transcript variant X2, mRNA	0.00	0.00	0.00	0.00	0.00	4.29
36	XM_022476048.1: putative uncharacterized protein DDB_G0279653 (LOC111129595), mRNA	0.00	0.00	0.00	0.00	0.00	2.50
37	XM_022488185.1: mucin-5AC-like (LOC111136981), transcript variant X2, mRNA	0.00	0.00	0.00	0.00	0.00	8.45

38	XR_002636999.1: uncharacterized LOC111116155 (LOC111116155), ncRNA	0.00	0.00	0.00	0.00	0.00	5.39
39	XM_022438641.1: uncharacterized LOC111104616 (LOC111104616), mRNA	0.00	0.00	0.00	-4.03	0.00	0.00
40	XM_022467830.1: mediator of RNA polymerase II transcription subunit 25-like (LOC111124695), transcript variant X3, mRNA	0.00	0.00	0.00	0.00	6.98	0.00
41	XR_002639827.1: uncharacterized LOC111137073 (LOC111137073), ncRNA	0.00	0.00	0.00	0.00	7.98	0.00
