# Supplementary material for: Exposure to plastic debris alters expression of biomineralization, immune, and stress-related genes in the eastern oyster (Crassostrea virginica)
Source: PLoS One. 2025 Apr 29;20(4):e0319165. doi: 10.1371/journal.pone.0319165 (PMC12040131; doi:10.1371/journal.pone.0319165)
Supplement: S3 Table — Positive log-fold changes are more highly expressed in the first group listed and negative log-fold changes are more highly expressed in the sex group listed. Non-significant comparisons are indicated as 0 log-fold change. (PDF) [file pone.0319165.s006.pdf]

	Gene	Male.Plastic.vs.Female.Shell	Male.Shell.vs.Female.Plastic	Female.Shell.vs.Female.Plastic	Male.Shell.vs.Male.Plastic	Male.Plastic.vs.Female.Plastic	Male.Shell.vs.Female.Shell
1	XR_002633972.1: uncharacterized LOC111100276 (LOC111100276), ncRNA	0.00	0.00	8.21	-8.55	8.55	-8.21
2	XM_022439393.1: uncharacterized LOC111105230 (LOC111105230), transcript variant X1, mRNA	0.00	-6.68	0.00	-9.04	0.00	-8.59
3	XM_022445765.1: uncharacterized LOC111109585 (LOC111109585), transcript variant X3, mRNA	5.90	0.00	-6.04	0.00	0.00	7.04
4	XM_022479699.1: phosphatidylinositol 3,4,5-trisphosphate 5-phosphatase 2A-like (LOC111132057), transcript variant X4, mRNA	8.16	0.00	-7.72	0.00	0.00	8.29
5	XM_022489306.1: uncharacterized LOC111137705 (LOC111137705), transcript variant X2, mRNA	0.00	-4.93	0.00	-6.61	0.00	-6.83
6	XR_002634969.1: uncharacterized LOC111104807 (LOC111104807), transcript variant X2, ncRNA	0.00	5.32	0.00	10.36	0.00	6.54
7	XM_022452159.1: protein YIPF3-like (LOC111113833), transcript variant X1, mRNA	-8.24	0.00	0.00	10.46	-9.84	0.00
8	XM_022455267.1: phytochrome-interacting ankyrin-repeat protein 1-like (LOC111116050), transcript variant X4, mRNA	0.00	7.34	8.31	0.00	6.89	0.00
	XM_022466730.1: mediator of RNA polymerase II						

http://localhost:12179/session/file3be91e68dfd0.html[11/13/23, 11:05:51 AM]

9	transcription subunit 7-like (LOC111123965), transcript variant X1, mRNA	-7.00	0.00	0.00	9.02	-10.43	0.00
10	XM_022485445.1: vesicle-associated membrane protein 3-like (LOC111135408), transcript variant X1, mRNA	0.00	7.31	7.72	0.00	9.17	0.00
11	XR_002636045.1: uncharacterized LOC111111513 (LOC111111513), ncRNA	-8.91	0.00	0.00	8.44	-11.01	0.00
12	XM_022446094.1: inhibitor of growth protein 3-like (LOC111109845), transcript variant X1, mRNA	0.00	0.00	-7.94	0.00	0.00	6.28
13	XM_022468895.1: uncharacterized LOC111125286 (LOC111125286), transcript variant X1, mRNA	0.00	0.00	0.00	0.00	8.83	-8.31
14	XM_022483964.1: flotillin-2a-like (LOC111134688), transcript variant X1, mRNA	0.00	0.00	0.00	-8.46	0.00	-7.87
15	XM_022438641.1: uncharacterized LOC111104616 (LOC111104616), mRNA	0.00	0.00	0.00	-4.18	2.32	0.00
16	XM_022442828.1: uncharacterized LOC111107567 (LOC111107567), transcript variant X1, mRNA	0.00	0.00	12.30	0.00	8.88	0.00
17	XM_022432886.1: uncharacterized LOC111100790 (LOC111100790), transcript variant X1, mRNA	0.00	0.00	0.00	0.00	0.00	-8.46
	XM_022437610.1: E3 ubiquitin-protein ligase rnf213-alpha-						

18	like (LOC111103955), mRNA	0.00	0.00	0.00	0.00	0.00	2.89
19	XM_022449961.1: uncharacterized LOC111112448 (LOC111112448), transcript variant X2, mRNA	0.00	0.00	0.00	0.00	0.00	4.29
20	XM_022453094.1: serine-rich adhesin for platelets-like (LOC111114676), transcript variant X2, mRNA	0.00	0.00	0.00	0.00	0.00	6.84
21	XM_022469284.1: uncharacterized LOC111125453 (LOC111125453), transcript variant X1, mRNA	0.00	0.00	0.00	0.00	0.00	-8.24
22	XR_002639194.1: uncharacterized LOC111133109 (LOC111133109), transcript variant X2, ncRNA	0.00	0.00	0.00	0.00	0.00	9.02
23	XM_022439842.1: runt-related transcription factor 1-like (LOC111105509), transcript variant X2, mRNA	0.00	0.00	0.00	0.00	-6.60	0.00
24	XM_022440902.1: uncharacterized LOC111106289 (LOC111106289), mRNA	0.00	0.00	0.00	0.00	-6.78	0.00
25	XM_022449646.1: uncharacterized LOC111112232 (LOC111112232), transcript variant X3, mRNA	0.00	0.00	0.00	0.00	-8.56	0.00
26	XM_022483641.1: uncharacterized LOC111134527 (LOC111134527), transcript variant X2, mRNA	0.00	0.00	0.00	0.00	-5.06	0.00
	XR_002639827.1: uncharacterized						

http://localhost:12179/session/file3be91e68dfd0.html[11/13/23, 11:05:51 AM]

27	LOC111137073 (LOC111137073), ncRNA	0.00	0.00	0.00	0.00	8.28	0.00
