# Supplementary figures and images for: Exposure to plastic debris alters expression of biomineralization, immune, and stress-related genes in the eastern oyster (Crassostrea virginica)
Source: PLoS One. 2025 Apr 29;20(4):e0319165. doi: 10.1371/journal.pone.0319165 (PMC12040131; doi:10.1371/journal.pone.0319165)

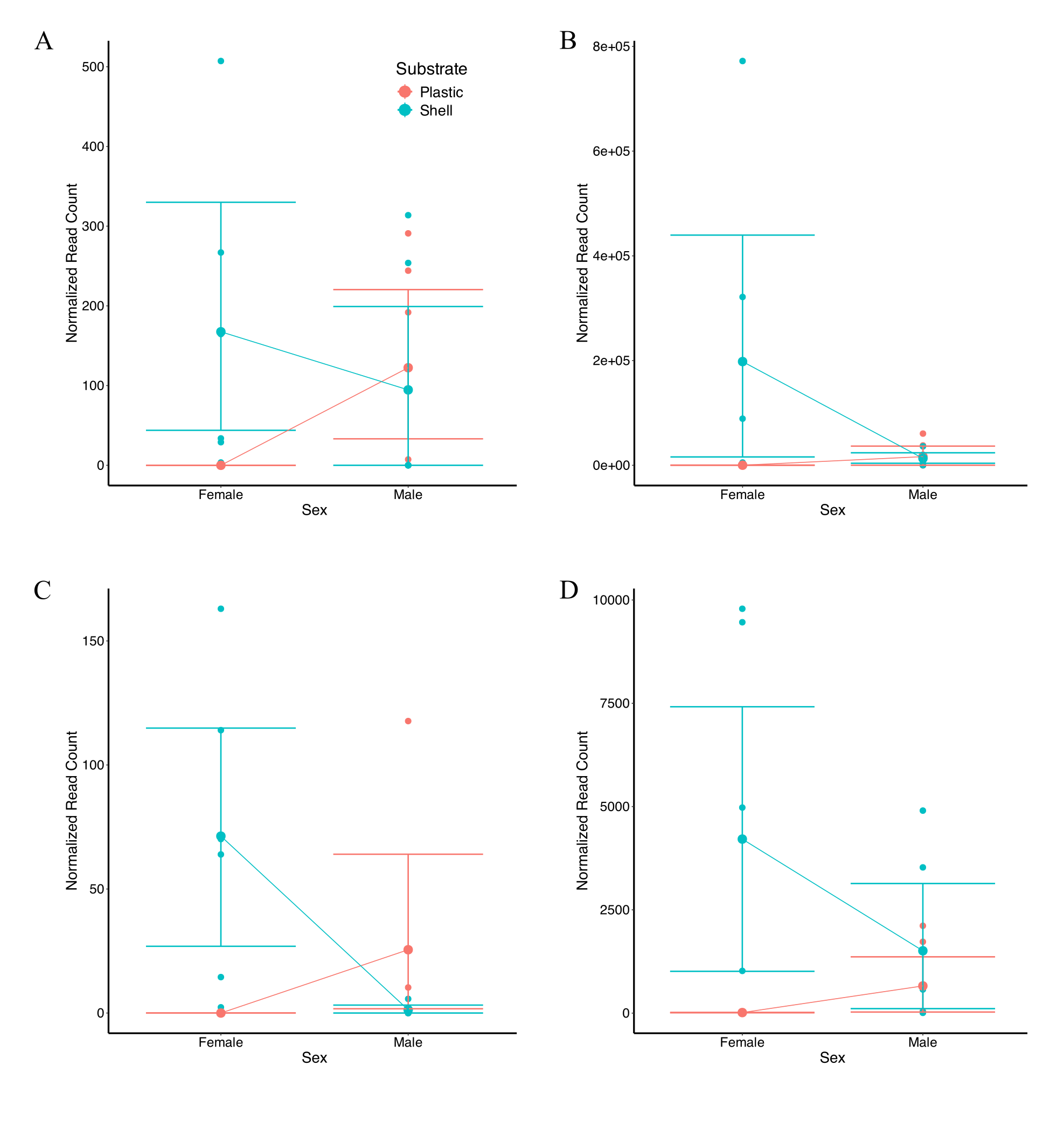

Supplement: S1 Fig — Norm of reaction for the four genes differentially expressed in gill tissue in response to all three model factors: sex, substrate, and sex-by-substrate (A) XM_022448393.1: Multimerin-1-like (LOC111111409), transcript variant X2, (B) XM_022476860.1: Fibroin heavy chain-like (LOC111130129) (C) XR_002639791.1: uncharacterized (LOC111136835) (D) XM_022454702.1: mantle protein-like (LOC111115840). Small points are individual read counts and large points are mean read counts with 95% confidence interval for each group of oysters with lines connecting means between sexes of the same substrate. (TIF) [file pone.0319165.s007.tif]

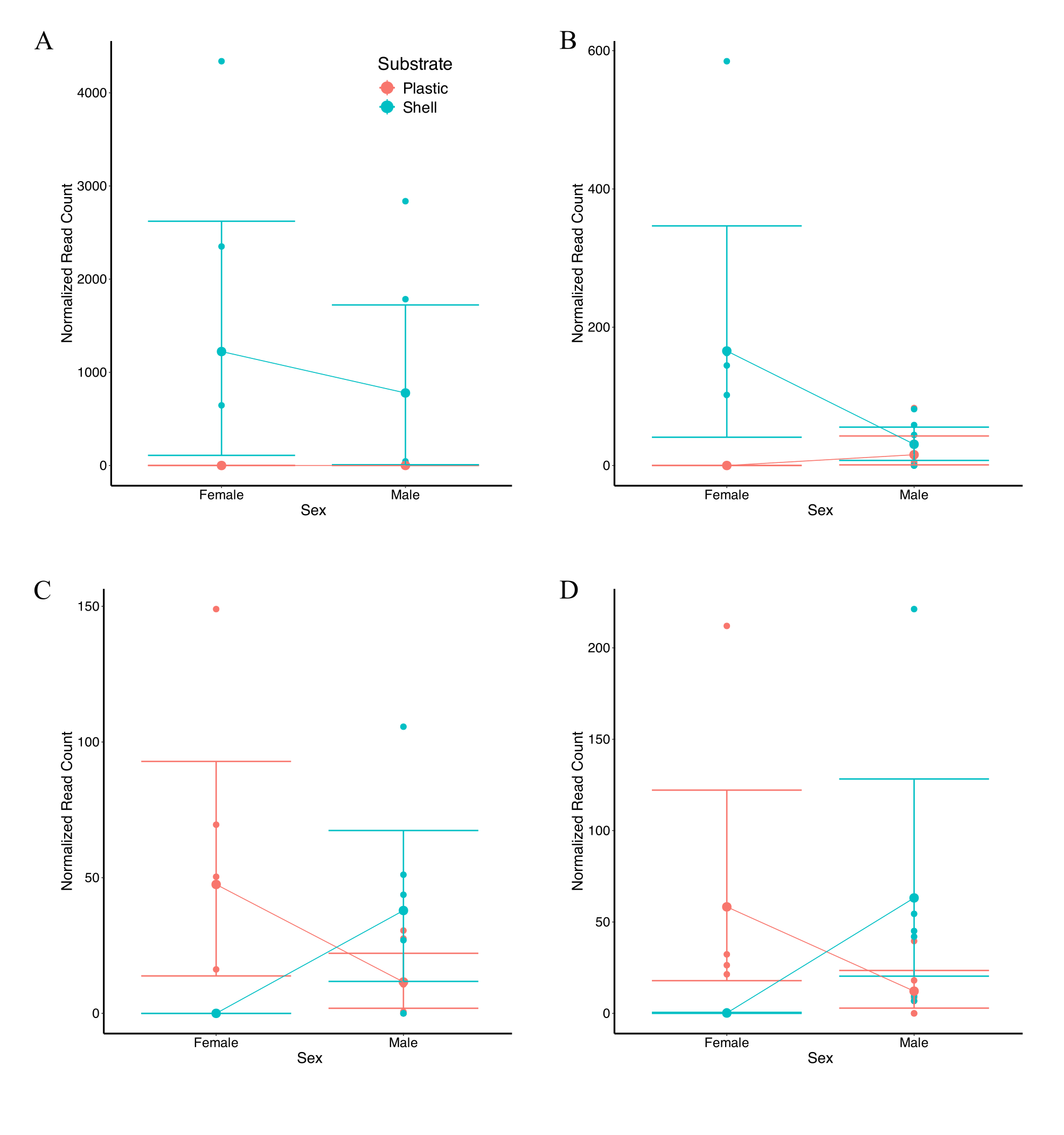

Supplement: S2 Fig — Norm of reaction for four representative genes differentially expressed in gill tissue in response to substrate with (A) and (B) representing genes more highly expressed on shell and (C) and (D) representing genes more highly expressed on plastic: (A) XM_022471172.1: uncharacterized protein K04H4.2-like (LOC111126488), (B) XM_022471207.1: uncharacterized (LOC111126514), (C) XM_022460099.1: pumilio homolog 3-like (LOC111119682), transcript variant X3, (D) XR_002637717.1: uncharacterized (LOC111120970). Small points are individual read counts and large points are mean read counts with 95% confidence interval for each group of oysters with lines connecting means between sexes of the same substrate. (TIF) [file pone.0319165.s008.tif]

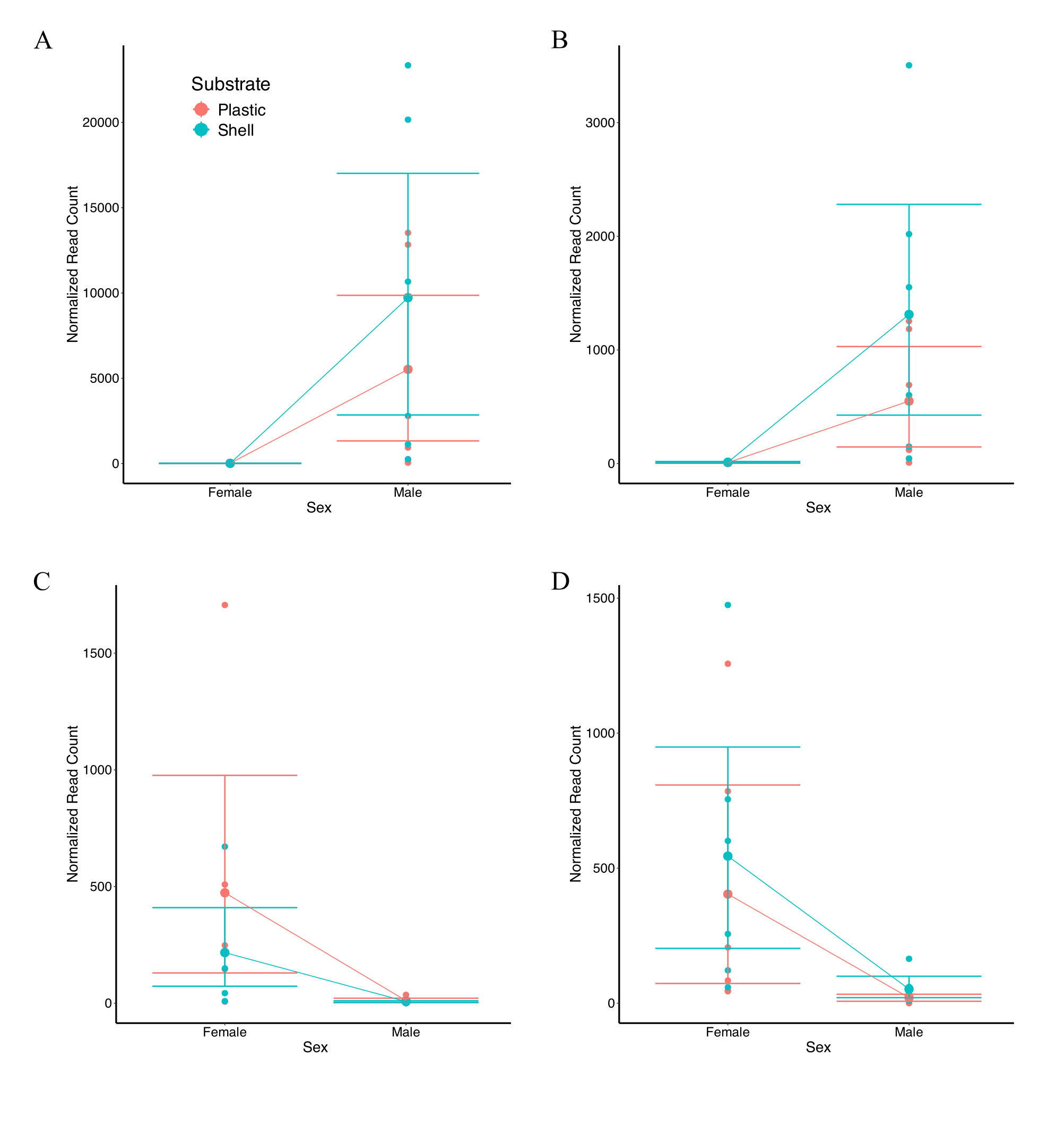

Supplement: S3 Fig — Norm of reaction for four representative genes differentially expressed in gonad tissue in response to sex with (A) and (B) representing genes more highly expressed in males regardless of substrate and (C) and (D) representing genes more highly expressed in females regardless of substrate: (A) XM_022471154.1: spindle assembly checkpoint kinase-like (LOC111126472), (B) XM_022461657.1: testis-specific serine/threonine-protein kinase 1-like (LOC111120728), (C) XM_022461132.1: cell wall protein DAN4-like (LOC111120418), (D) XM_022481064.1: phosphoethanolamine N-methyltransferase 3-like (LOC111133025), transcript variant X3. Small points are individual read counts and large points are mean read counts with 95% confidence interval for each group of oysters with lines connecting means between sexes of the same substrate. (TIF) [file pone.0319165.s009.tif]

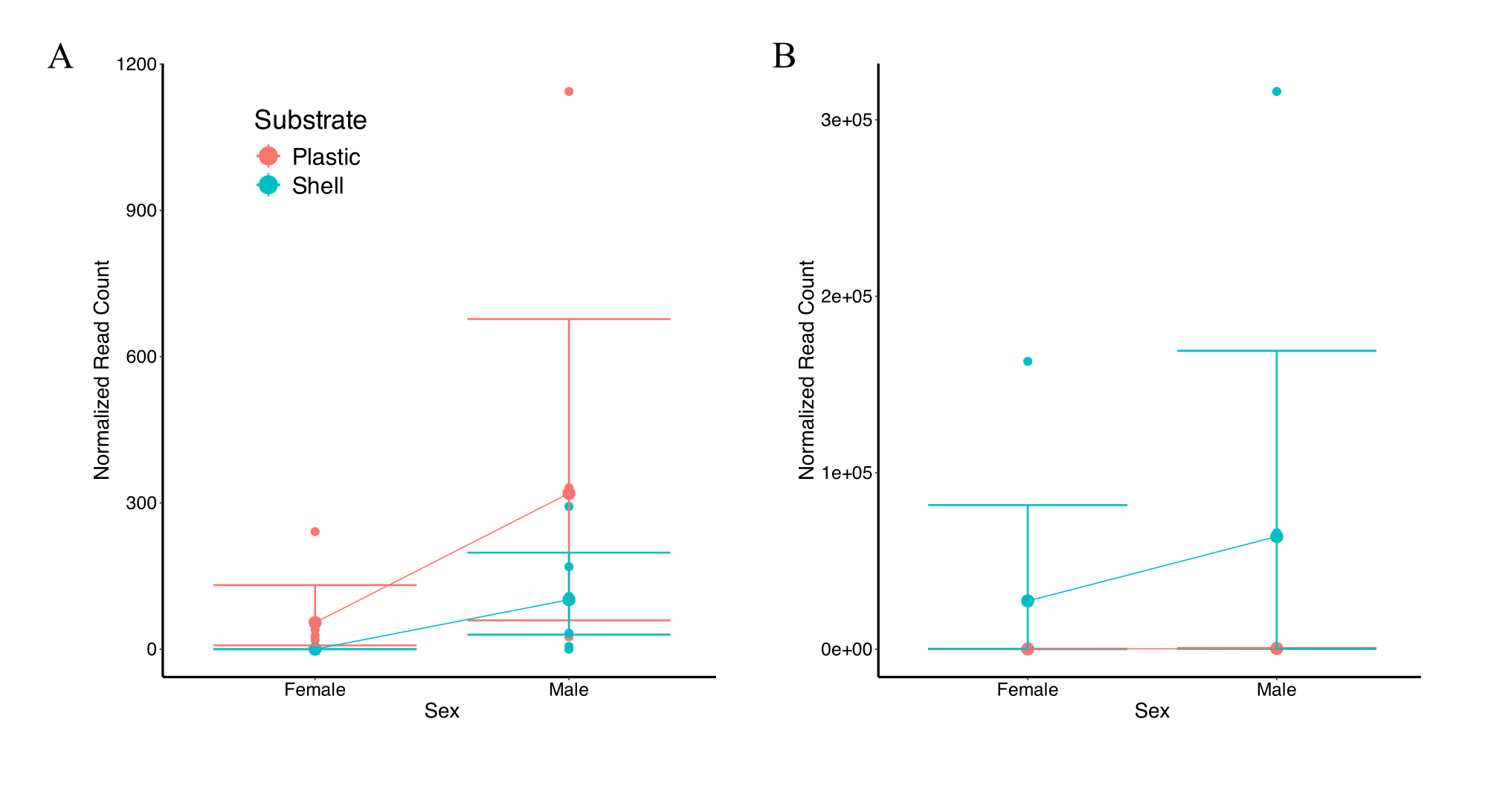

Supplement: S4 Fig — Norm of reaction for four representative genes differentially expressed in gonad tissue in response to substrate with (A) representing genes more highly expressed on plastic and (B) representing genes more highly expressed on shell: (A) XM_022457216.1: cell wall integrity and stress response component 4-like (LOC111117955), transcript variant X2 and (B) XM_022489166.1: ctenidin-1-like (LOC111137625). Small points are individual read counts and large points are mean read counts with 95% confidence interval for each group of oysters with lines connecting means between sexes of the same substrate. (TIFF) [file pone.0319165.s010.tiff]

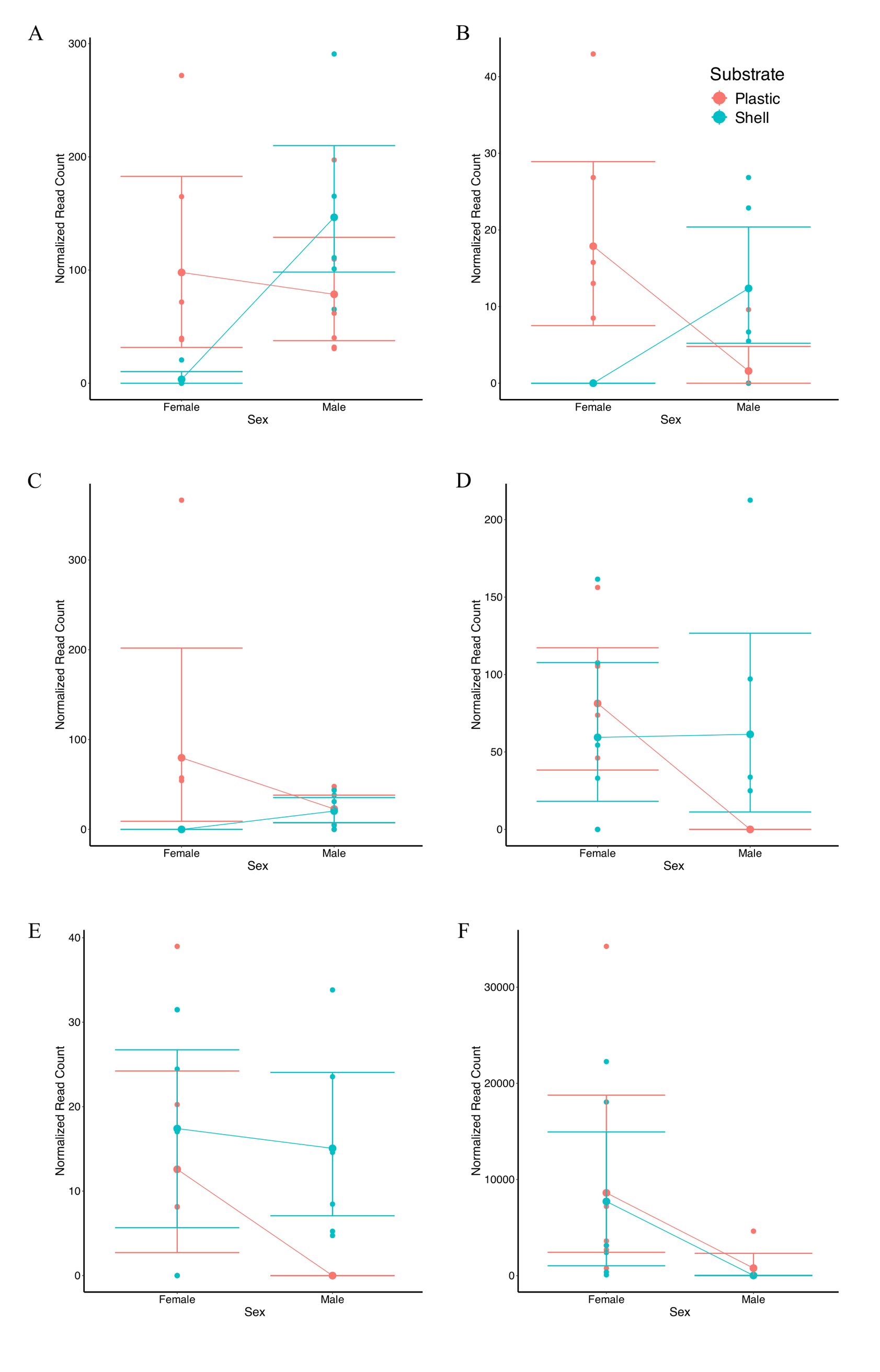

Supplement: S5 Fig — Norm of reaction for five representative genes differentially expressed in gonad tissue in response to sex-by-substrate: (A)XM_022435151.1: CUGBP Elav-like family member 2 (LOC111102426), transcript variant X20, (B) XM_022446094.1: inhibitor of growth protein 3-like (LOC111109845), transcript variant X1, (C) XM_022458260.1: syntenin-1-like (LOC111118686), transcript variant X1, (D) XR_002635236.1: uncharacterized (LOC111106591), (E) XM_022458233.1: uncharacterized (LOC111118638), and (F) XM_022461020.1: vitellogenin-like (LOC111120293). Small points are individual read counts and large points are mean read counts with 95% confidence interval for each group of oysters with lines connecting means between sexes of the same substrate. (TIF) [file pone.0319165.s011.tif]
